# Fermentative aminopyrrolnitrin production by metabolically engineered *Corynebacterium glutamicum*

**DOI:** 10.1186/s12934-024-02424-y

**Published:** 2024-05-23

**Authors:** Virginia Ryandini Melati Putri, Min-Hee Jung, Ji-Young Lee, Mi-Hyang Kwak, Theavita Chatarina Mariyes, Anastasia Kerbs, Volker F. Wendisch, Hee Jeong Kong, Young-Ok Kim, Jin-Ho Lee

**Affiliations:** 1 Department of Food Science & Biotechnology, BB21+, Kyungsung University, Busan, 48434 Republic of Korea; 2 Faculty of Biology and Center for Biotechnology, Bielefeld University, Bielefeld, Germany; 3 Biotechnology Research Division, National Institute of Fisheries Science, Busan, 46083 Republic of Korea

**Keywords:** Aminopyrrolnitrin, Mutant TrpD, 7-Chloro-l-tryptophan, Monodechloroaminopyrrolnitrin, Microbial production, *Corynebacterium glutamicum*

## Abstract

Aminopyrrolnitrin (APRN), a natural halogenated phenylpyrrole derivative (HPD), has strong antifungal and antiparasitic activities. Additionally, it showed 2.8-fold increased photostability compared to pyrrolnitrin, a commercially available HPD with antimicrobial activity. For microbial production of APRN, we first engineered anthranilate phosphoribosyltransferase encoded by *trpD* from *Corynebacterium glutamicum*, resulting in a TrpD^A162D^ mutation that exhibits feedback-resistant against l-tryptophan and higher substrate affinity compared to wild-type TrpD. Plasmid-borne expression of *trpD*^A162D^ in *C. glutamicum* TP851 strain with two copies of *trpD*^A162D^ in the genome led to the production of 3.1 g/L l-tryptophan in flask culture. Subsequent step for l-tryptophan chlorination into 7-chloro-l-tryptophan was achieved by introducing diverse sources of genes encoding tryptophan 7-halogenase (PrnA or RebH) and flavin reductase (Fre, PrnF, or RebF). The combined expression of *prnA* from *Serratia grimesii* or *Serratia plymuthica* with flavin reductase gene from *Escherichia coli*, *Pseudomonas fluorescens*, or *Lechevalieria aerocolonigenes* yielded higher production of 7-chloro-l-tryptophan in comparison to other sets of two-component systems. In the next step, production of putative monodechloroaminopyrrolnitrin (MDAP) from 7-chloro-l-tryptophan was achieved through the expression of *prnB* encoding MDAP synthase from *S. plymuthica* or *P. fluorescens*. Finally, an artificial APRN biosynthetic pathway was constructed by simultaneously expressing genes coding for tryptophan 7-halogenase, flavin reductase, MDAP synthase, and MDAP halogenase (PrnC) from different microbial sources within the l-tryptophan-producing TP851 strain. As *prnC* from *S. grimesii* or *S. plymuthica* was introduced into the host strain, which carried plasmids expressing *prnA* from *S. plymuthica*, *fre* from *E. coli*, and *prnB* from *S. plymuthica*, APN3639 and APN3638 accumulated 29.5 mg/L and 28.1 mg/L of APRN in the culture broth. This study represents the first report on the fermentative APRN production by metabolically engineered *C. glutamicum*.

**Supplementary Information:**

The online version contains supplementary material available at 10.1186/s12934-024-02424-y.

## Introduction

**Fig. 1:**
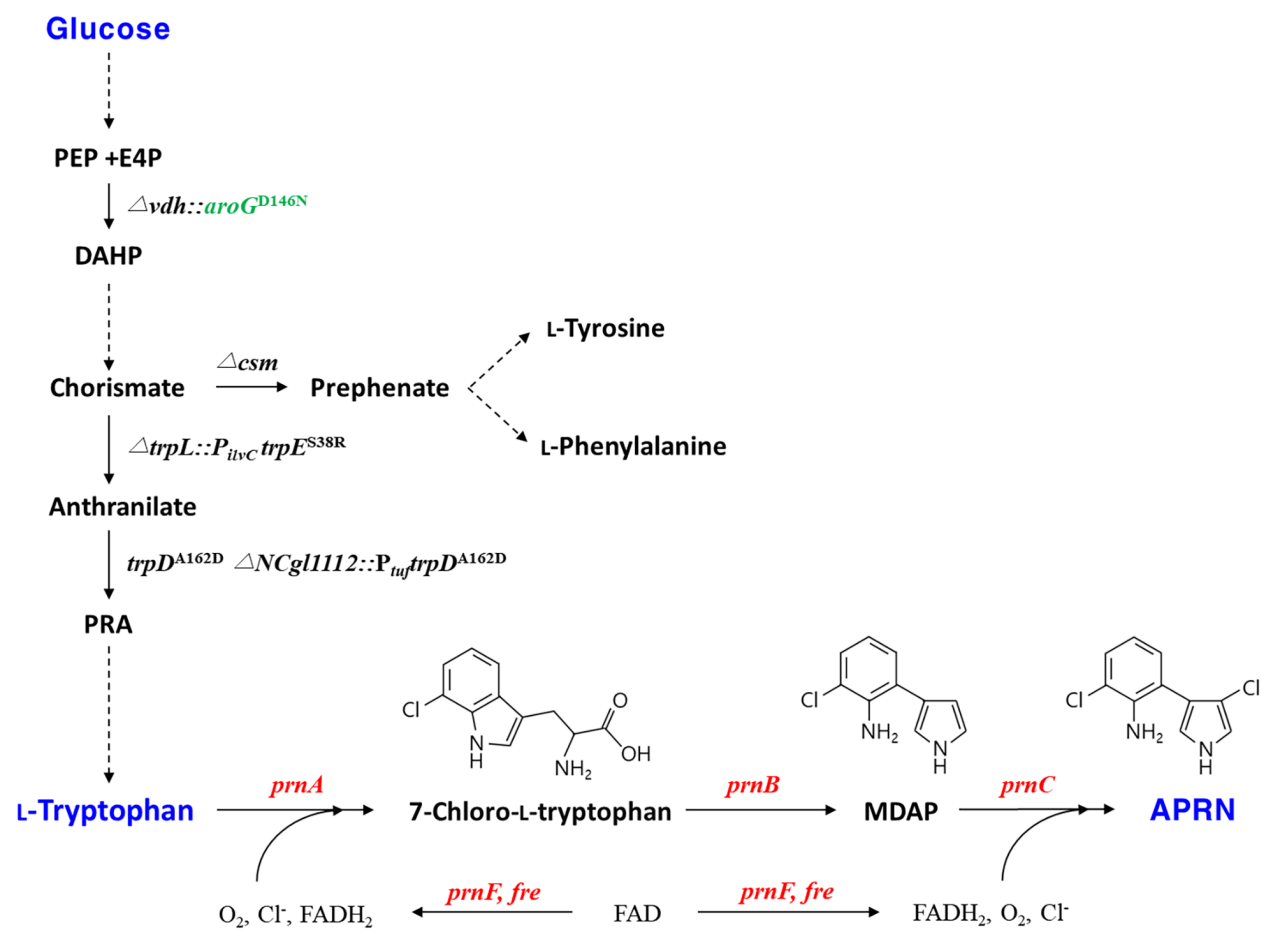
Schematic representation of metabolically engineered *C. glutamicum* producing aminopyrrolnitrin. Genes depicted in red and green, respectively, indicate plasmid- and genome-based heterologous expressions. Dashed arrows show several reaction steps. The abbreviations are as follows: PEP, phosphoenolpyruvate; E4P, erythrose 4-phosphate; DAHP, 3-deoxy-D-arabinoheptulosonate-7-phosphate; PRA, phosphoribosyl anthranilate; MDAP, monodechloroaminopyrrolnitrin; APRN, aminopyrrolnitrin. Genes and correspoding enzymes are as follows: *vdh*, vanillin dehydrogenase; *aroG*, DAHP synthase from *E. coli*; *trpL*, tryptophan operon leader peptide; *trpE*, anthranilate synthase; *trpD*, anthranilate phosphoribosyltransferase; *prnA*/*rebH*, tryptophan 7-halogenase; *prnB*, MDAP synthase; *prnC*, MDAP halogenase; *prnF*/*fre*/*rebF*, flavin reductase

Pyrrolnitrin (3-chloro-4-(3-chloro-2-nitrophenyl)pyrrole, PRN, C_10_H_6_Cl_2_N_2_O_2_) is a phenylpyrrole derivative containing two chlorine atoms and one nitro group [42]. In microbes, PRN biosynthesis involves a series of four enzymatic steps from l-tryptophan [25] (Fig. 1). The flavin-dependent tryptophan 7-halogenase encoded by *prnA* or *rebH* is responsible for converting L-tryptophan into 7-chloro-l-tryptophan (7-Cl-Trp), exhibiting regioselective chlorination at the 7-carbon of l-tryptophan [10, 62]. Next, monodechloroaminopyrrolnitrin (MDAP) synthase encoded by *prnB* catalyzes the rearrangement and decarboxylation of 7-Cl-Trp to yield MDAP [7]. Then, the chlorination of MDAP by MDAP halogenase encoded by *prnC* produces aminopyrrolnitrin (APRN), followed by oxidation of the amino group of APRN by APRN oxygenase encoded by *prnD* to form PRN [30]. The initial, third, and final steps of PRN biosynthesis require FADH_2_ provided by flavin reductase [31]. The gene cluster in PRN biosynthetic pathway is organized as *prnABCD* operon in *Pseudomonas fluorescens*, *P. protegenes*, *Burkholderia contamins*, *Serratia plymuthica*, *S. grimesii*, etc [14, 34, 37]. Microbial PRN production has been identified in many screened wild-type strains, i.a., *P. pyrrocinia, P. fluorescens, P. protegens, P. chlororaphis, Burkholderia cepacia, Enterobacter agglomerans*, and *Serratia plymuthica* [5, 34, 42, 45, 52]. Despite examinations of their genetic regulations and biochemical studies, there are few reports on strain engineering and media optimization for PRN production [34, 42].

PRN shows a broad spectrum of antimicrobial activities against a wide range of bacteria, yeasts, and fungi, such as *Staphylococcus aureus, Mycobacterium tuberculosis, Streptomyces antibioticus, Ustilago maydis, Candida albicans, Trichophyton asteroids, Sporotrichum schenckii, Sclerotinia sclerotiorum*, *Botrytis cinerea*, *Rhizoctonia solani*, *Fusarium sambucinum*, *F. graminearum*, and *Pythium aphanidermatum* [16, 42, 55]. Thus, it not only plays a significant role in the biocontrol of plant diseases caused by soil-borne and foliar fungal pathogens [34], but also serves as a key ingredient in drugs for the treatment of superficial dermatophytic fungal infections [55]. It also displays nematocidal and repellent activity against *Caenorhabditis elegans* [36]. On the other hand, PRN applications are limited due to the poor photostability when exposed to sunlight, resulting in the loss of antifungal activities [26, 46]. Recently, we represented that APRN, a precursor of PRN, also has strong antifungal activities against *Candida albicans, C. palmioleophila, C. zeylaroides, Bipolaris californica, Fusarium proliferatum, Trichosporon cavemicola, Debaryomyces hansenii*, and *Wickerhamomyces anomalus* as well as anti-parasitic activity against *Uronema marinum* [24]. Furthermore, APRN was found to act as a non-steroidal androgen-receptor antagonist [15]. In this context, APRN may stand out as a promising candidate for developing novel fungicides, antiparasitic agents, or androgen-receptor antagonists that are less toxic and environmentally friendly.

*Corynebacterium glutamicum* is the workhorse for the production of a plethora of amino acids, amines, alcohols, organic acids, and aromatic compounds [28, 29, 60, 61]. l-Tryptophan production in *C. glutamicum* was enabled by plasmid-borne expression of *trpD* from *Escherichia coli* and genome-based expression of both *aroG*^FBR^ from *E. coli* and *trpE*^FBR^, together with the deletion of *csm* encoding chorismate mutase [57] (Fig. 1). Lately, this bacterium has been employed to produce l-tryptophan precursors and derivatives, e.g., *N*-methylanthranilate, indole, and indole 3-acetic acid [23, 35, 59], along with halogenated l-tryptophans and their derivatives, e.g., 7-Cl-Trp, 7-bromo-l-tryptophan, 7-Cl-indole, 7-Br-indole, and 7-Br-tryptamine [20, 56, 57]. The production of APRN precursor, 7-Cl-Trp, was established by introducing robust chlorinating tryptophan 7-halogenase and flavin reductase encoded by *rebH* and *rebF* from *Lechevalieria aerocolonigenes* in l-tryptophan-producing *C. glutamicum*, yielding 108 mg/L 7-Cl-Trp in flask cultures [57, 62].

Here, we first engineered anthranilate phosphoribosyltransferase encoded by *trpD* from *C. glutamicum* to increase the availability of l-tryptophan required for APRN biosynthesis. An artificial APRN biosynthetic pathway was then assembled through the combinatorial expression of genes coding for tryptophan 7-halogenase, MDAP synthase, MDAP halogenase, and flavin reductase from several microbial sources in engineered l-tryptophan-producing *C. glutamicum* (Fig. 1). Moreover, APRN presented higher photostability than PRN, suggesting its potential application as a novel biofungicide derived from halogenated phenylpyrroles. This is the first report on the fermentative APRN production by metabolically engineered *C. glutamicum*.

## Results

### Production of l-tryptophan by engineered feedback-resistant anthranilate phosphorybosyltransferase mutants

The *C. glutamicum* strain TP679, as reported previously, produced less than 0.05 g/L l-tryptophan, whereas it produced 3.24 g/L anthranilate as a primary product in flask culture [57]. This outcome is likely attributed to the feedback inhibition of anthranilate phosphoribosyltransferase encoded by *trpD* by the end-product l-tryptophan [40]. Although the A162E TrpD mutant in which l-alanine at the 162nd residue is substituted with l-glutamic acid represented feedback resistance against l-tryptophan or 5-methyltryptophan [40], a plasmid-borne expression of *trpD*^A162E^ in TP679 resulted in a significant production of anthranilate and l-tryptophan (Table 1). To increase l-trytophan titer and decrease anthranilate titer, in this study, the l-alanine residue was replaced with negatively charged amino acids, l-glutamate and l-aspartate as well as structurally similar and polar amino acids, l-serine and l-threonine, using site-directed mutagenesis, yielding plasmids pX-A162E, pX-A162D, pX-A162S, and pX-A162T, respectively. To evaluate the effect of TrpD mutations and to stably maintain the recombinant plasmids, constructed plasmids were subcloned into pCES208 and then transformed into TP679. In flask cultures, a plasmid-borne expression of wild-type *trpD* in TP679 resulted in accumulation of 3.3 g/L anthranilate and 0.1 g/L l-tryptophan (Table 1). Mutants A162S and A162T led to statistically significant but marginal increases of l-tryptophan titers. On the one hand, TP679 with either the mutant A162E or A162D showed significantly increased titers of l-tryptophan by 13- and 25-fold, respectively, while the anthranilate titers were significantly decreased compared to the control strain with A162. In particular, TP679 with A162D enabled production of 2.74 g/L l-tryptophan with little accumulation of anthranilate after 48 h cultivation in baffled flasks (Table 1).

**Table 1:** Production of l-tryptophan and anthranilate by recombinant *C. glutamicum* TP679 harboring a mutant *trpD* gene

Strain	Plasmid	OD_600nm_	ANT^*^ (g/L)	TRP^**^ (g/L)
TP679	-	40.0 ± 4.3	3.24 ± 0.52	< 0.05
	pC-A162	41.9 ± 6.2	3.32 ± 0.15	0.11 ± 0.04
	pC-A162D	42.9 ± 7.0	< 0.05	2.74 ± 0.29
	pC-A162E	38.7 ± 2.1	2.61 ± 0.50	1.48 ± 0.16
	pC-A162S	42.8 ± 6.1	3.30 ± 0.37	0.17 ± 0.02
	pC-A162T	39.6 ± 5.9	3.43 ± 0.56	0.27 ± 0.04
TP793	-	42.3 ± 2.0	2.93 ± 0.38	0.52 ± 0.09
TP851	-	43.2 ± 7.6	1.62 ± 0.45	1.39 ± 0.39
	pC-A162D	43.3 ± 4.3	< 0.05	3.11 ± 0.27

ANT^*^, anthranilate; TRP^**^, l-tryptophan

**Fig. 2:**
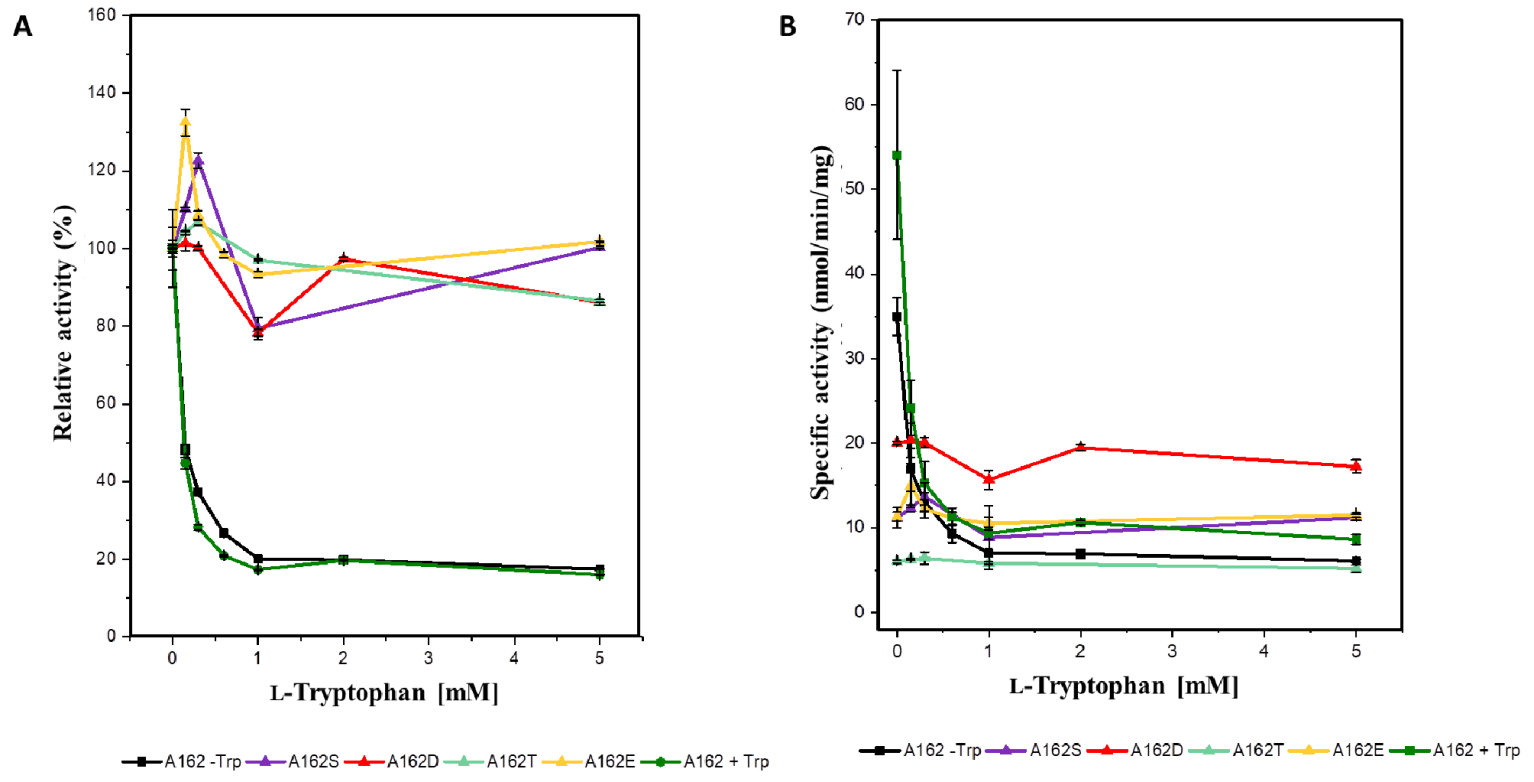
Relative inhibition profiles (**A**) and absolute activities (**B**) of wild-type and mutants TrpD by l-tryptophan. All strains were grown in LB medium with 50 mg/L l-tryptophan, including “A162 + Trp”, except for “A162 - Trp” which was grown without l-tryptophan

To evaluate the extent of feedback inhibition of TrpD mutants by l-tryptophan, we measured TrpD activity in *C. glutamicum* strain ATCC 13,032 harboring constructed plasmids. The inhibition profiles for both the wild-type and mutant TrpDs in response to l-tryptophan are shown in Fig. 2. Notably, wild-type TrpD only showed about 20% residual activity in the presence of 1 mM or more L-tryptophan, while the mutants A162S, A162T, A162E and A162D demonstrated feedback resistance, maintaining over 80% residual activity in the presence 1 mM or more L-tryptophan (Fig. 2A). As amino acid substitutions often impact the maximal velocity (*V*_max_), we also plotted the absolute activities (Fig. 2B). The feedback-resistant mutant A162T maintained a low activity of about 6 ± 0.2 nmol/min/mg protein, regardless of the absence or presence of L-tryptophan. In contrast, the tryptophan-sensitive wild-type TrpD showed higher activity, even in the presence of 5 mM L-tryptophan. Conversely, the feedback-resistant mutant A162D maintained an activity of 15 to 20 nmol/min/mg protein in the absence or presence of l-tryptophan. Notably, the activity of mutant A162D was about three-fold higher than that of wild-type TrpD in the presence of 0.4 to 5 mM l-tryptophan. Moreover, when the TrpD activity of crude extracts with either wild-type A162 or mutant A162D was determined with varying concentrations of the substrate anthranilate (0.1 to 1.6 µM), *K*_m_ values could be estimated. Whilst wild-type TrpD required 4.3 ± 0.6 µM anthranilate for half-maximal activity, mutant A162D showed higher substrate affinity as only 0.74 ± 0.3 µM anthranilate were required for half-maximal activity. These findings align with the observation that mutant A162D displayed the highest activity in the presence of up to 5 mM L-tryptophan, along with a higher substrate affinity. This consistency supports the conclusion that overexpression of the gene encoding TrpD mutant A162D had the most beneficial effect on L-tryptophan production, resulting in a pronounced decrease in the substrate anthranilate (Table 1).

Based on these results, chromosomal expression of *trpD*^A162D^ was chosen as next engineering step. TP793 was obtained by replacing the wild-type *trpD* on the genome with the mutant allele *trpD*^A162D^ using TP679 as the mother strain (Fig. S2A). In flask culture, this strain produced 2.93 g/L anthranilate and 0.52 g/L l-tryptophan, indicating that additional expression of *trpD*^A162D^ is required to reduce anthranilate and enhance l-tryptophan production (Table 1). To do this, TP851 strain with two copies of mutant *trpD*^A162D^ was constructed by inserting P_*tuf*_-*trpD*^A162D^ into the *NCgl1112*-deleted locus (Fig. S2B). *C. glutamicum* TP851 allowed the accumulation of 1.62 g/L anthranilate and 1.39 g/L l-tryptophan, while the plasmid-borne expression of *trpD*^A162D^ in TP851 led to the production of 3.11 g/L l-tryptophan in the flask culture after 48 h (Table 1). Accordingly, we employed TP851 as a mother strain for APRN production by *C. glutamicum*, even though it requires the expression of extra copies of *trpD*^A162D^ allele in genome for overprodcution of l-tryptophan and less accumulation of anthranilate.

### Comparison of photostability of APRN and PRN

Since both APRN and PRN have broad antimicrobial activities and diverse applications, we compared photostability of APRN and PRN dissolved in aprotic acetonitrile solvent. APRN had 2.8 times higher photostability than PRN when exposed to UV light for 60 min (Fig. S3). Accordingly, development of engineered *C. glutamicum* producing APRN instead of PRN using l-tryptophan-producing *C. glutamicum* as a base strain is considered a promising approach.

### Production of 7-chloro-l-tryptophan by combinatorial expression of tryptophan 7-halogenase and flavin reductase genes

**Fig. 3:**
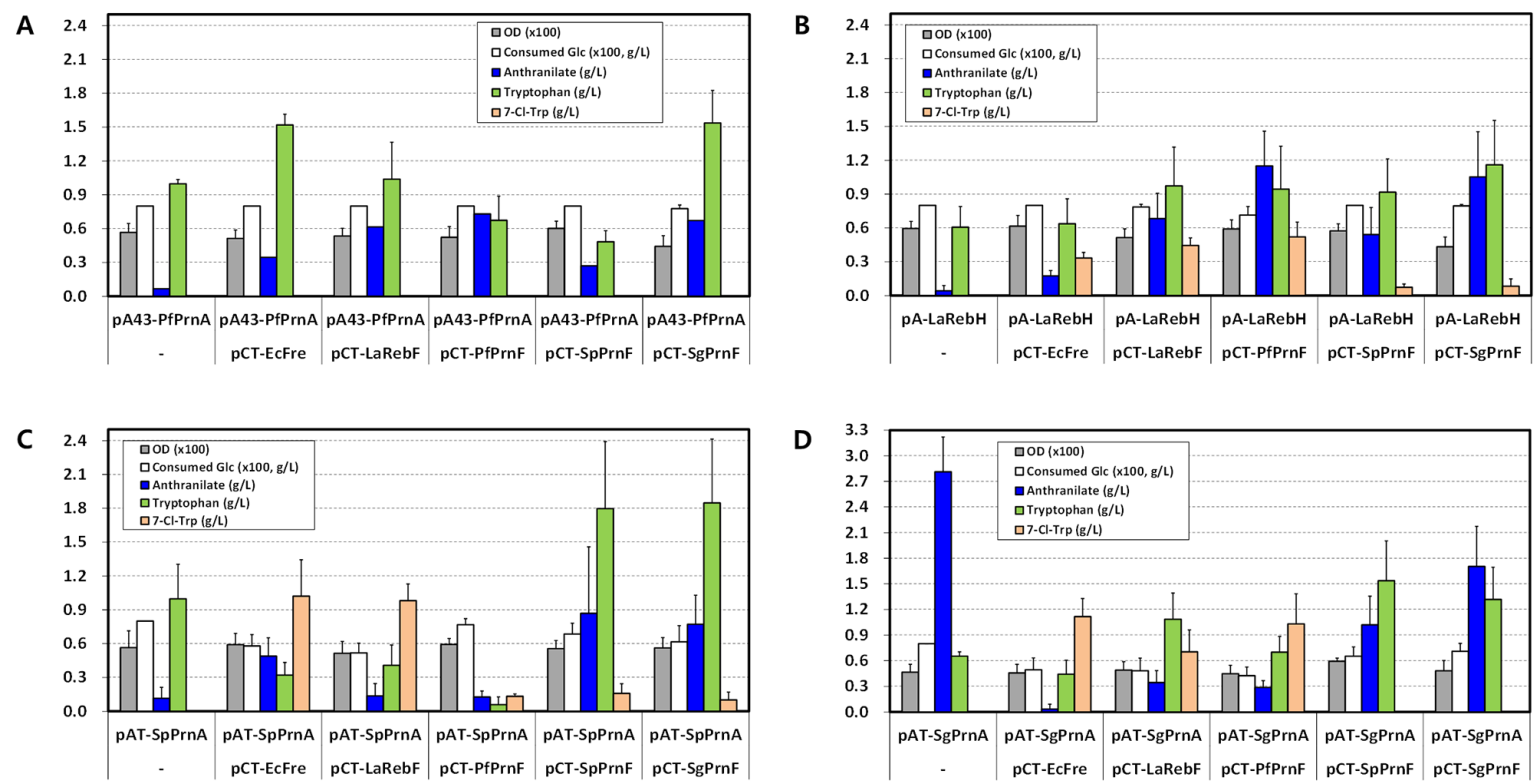
Production of 7-chloro-l-tryptophan and its precursors by recombinant *C. glutamicum* expressing different sets of genes encoding tryptophan 7-halogenase and flavin reductase in flask cultures. (**A**) Combinatorial expression of *prnA* from *P. fluorescens* with five different flavin reductase genes; (**B**) combinatorial expression of *rebH* from *L. aerocolonigenes* with five different flavin reductase genes; (**C**) combinatorial expression of *prnA* from *S. plymuthica* with five different flavin reductase genes; (**D**) combinatorial expression of *prnA* from *S. grimesii* with five different flavin reductase genes. Cells were grown in a flask fermentation medium supplemented with 50 mM CaCl_2_ for 48 h at 32 °C with vigorous shaking at 240 rpm. Mean and standard deviation of triplicate cultivation are given

As 7-Cl-Trp biosynthesis requires both tryptophan 7-halogenase and flavin reductase (Fig. 1), candidate genes encoding tryptophan 7-halogenase, including *rebH* from *L. aerocolonigenes* (*Lc-rebH*) and *prnA*s from *P. fluorescens* (*Pf-prnA*), *S. plymuthica* G3 (*Sp-prnA*), and *S. grimesii* (*Sg-prnA*), were mined through literature searches [25, 34, 37, 62]. For bacterial flavin reductases, three functionally identified genes were enlisted, i.e., *fre* from *E. coli* (*Ec-fre*), *rebF* from *L. aerocolonigenes* (*La-rebF*), and *prnF* from *P. fluorescens* Pf-5 (*Pf-prnF*) [31, 51, 62]. Moreover, putative *prnF* genes from *S. plymuthica* (*Sp-prnF*) and *S. grimesii* (*Sg-prnF*) were chosen based on sequence homology search using GenBank accession numbers KY867430 and KY867431 [43]. After optimizing the codon usage for *C. glutamicum*, the selected genes were synthesized and cloned into expression vectors of *C. glutamicum*. SDS-PAGE analysis using crude extracts of ATCC 13032 expressing tryptophan 7-halogenase genes showed a prominent *Sp*-PrnA protein band, followed by a slight increase of *Sg*-PrnA band, but no discernible *La*-RebH band (Fig. S4). A little expression of *Pf*-*prnA* was observed under control of the *ilvC*-M1 promoter [27]. Considering these results, we selected the plasmids pX43-PfPrnA, pX-LaRebH, pXT-SpPrnA, and pXT-SgPrnA as gene sources for tryptophan 7-halogenase. In the case of (putative) flavin reductases, Fre protein was produced very well in *C. glutamicum* (Fig. S5). *La*-RebF and *Sg*-PrnF were slightly produced, whereas PrnFs from *P. fluorescens* and *S. plymuthica* seemed to be poorly produced in *C. glutamicum*. After establishing combinatorial expression of different sets of genes in a two-component halogenase/reductase system [62], we evaluated the ability of assembled strains to produce 7-Cl-Trp in flask cultures. Recombinant TP851 strains containing a subset of *Pf-prnA*, each with five different flavin reductase genes, didn’t produce 7-Cl-Trp (Fig. 3A), suggesting that Pf-PrnA probably does not functionally work in *C. glutamicum*. Of the *La-rebH* subsets with five flavin reductase genes, the combined expression of *La-rebH*/*Pf-prnF* in TP851 (named CT28) resulted in production of 0.52 g/L of 7-Cl-Trp using fermentation medium with 80 g/L glucose and 50 mM CaCl_2_ in flask cultures (Fig. 3B). In the case of *Sp-prnA* subsets, the expression subsets *Sp-prnA*/*Ec-fre* (named CT36) and *Sp-prnA*/*La-rebF* (named CT37), respectively, enabled the production of 1.02 g/L and 0.98 g/L of 7-Cl-Trp (Fig. 3C). For the *Sg-prnA* subsets, the expression subsets *Sg-prnA*/*Ec-fre* and *Sg-prnA*/*Pf-prnF* in TP851 (named CT46 and CT48), respectively, allowed to the production of 1.12 g/L and 1.03 g/L of 7-Cl-Trp (Fig. 3D). However, a high accumulation of 7-Cl-Trp in the strain CT46 or CT36 led to retarded glucose consumption compared to the control strain, which is probably due to the cytotoxicity of 7-Cl-Trp toward *C. glutamicum* [57]. Taken together, the evaluated 7-Cl-Trp production titers among the expression subsets of the two-component system were the highest in TP851 harboring *Sg-prnA*/*Ec-fre*, followed by *Sg-prnA*/*Pf-prnF*, *Sp-prnA*/*Ec-fre*, and *Sp-prnA*/*La-rebF* (Fig. S8). In terms of 7-Cl-Trp production, tryptophan 7-halogenases from *S. plymuthica* and *S. grimesii* combined with flavin reductases from *E. coli*, *P. fluorescens*, and *L. aerocolonigenes* were demonstrated to function efficiently in *C. glutamicum*.

### Production of APRN in engineered *C. glutamicum* by assembling APRN biosynthetic pathway genes

With the aim of extending tryptophan biosynthetic pathway to APRN production *via* MDAP in *C. glutamicum* (Fig. 1), we adopted several microbial sources of *prnB*s and *prnC*s from *P. fluorescens* BL915 (*Pf*-*prnB* and *Pf*-*prnC*), *B. contamins* (*Bc-prnB* and *Bc-prnC*), *S. plymuthica* PR1-2 C (*Sp-prnB* and *Sp-prnC*), and *S. grimesii* (*Sg-prnB* and *Sg-prnC*) based on literature searches and bioinformatic analysis [8, 34, 42]. The selected genes were synthesized and cloned into the expression vector pCXE50T, and the resulting vectors were transformed into ATCC 13,032. According to analysis of protein production by SDS-PAGE, there were no significant differences in PrnB bands corresponding to the estimated molecular weight of 40 kDa from *B. contamins, P. fluorescens*, and *S. grimesii* compared to the control strain (Fig. S6, lanes 2, 4, 7), whereas *Sp*-PrnB was well produced in *C. glutamicum* (Fig. S6, lane 5). On the one hand, the differentiated PrnC bands (estimated molecular weight of 65 kDa) from *P. fluorescens*, *B. contamins, S. plymuthica* and *S. grimesii* could not be observed (Fig. S7). Accordingly, we cloned diverse sources of *prnB* and *prnC* genes and confirmed that the PrnB from *S. plymuthica* showed prominent production in *C. glutamicum.*

**Fig. 4:**
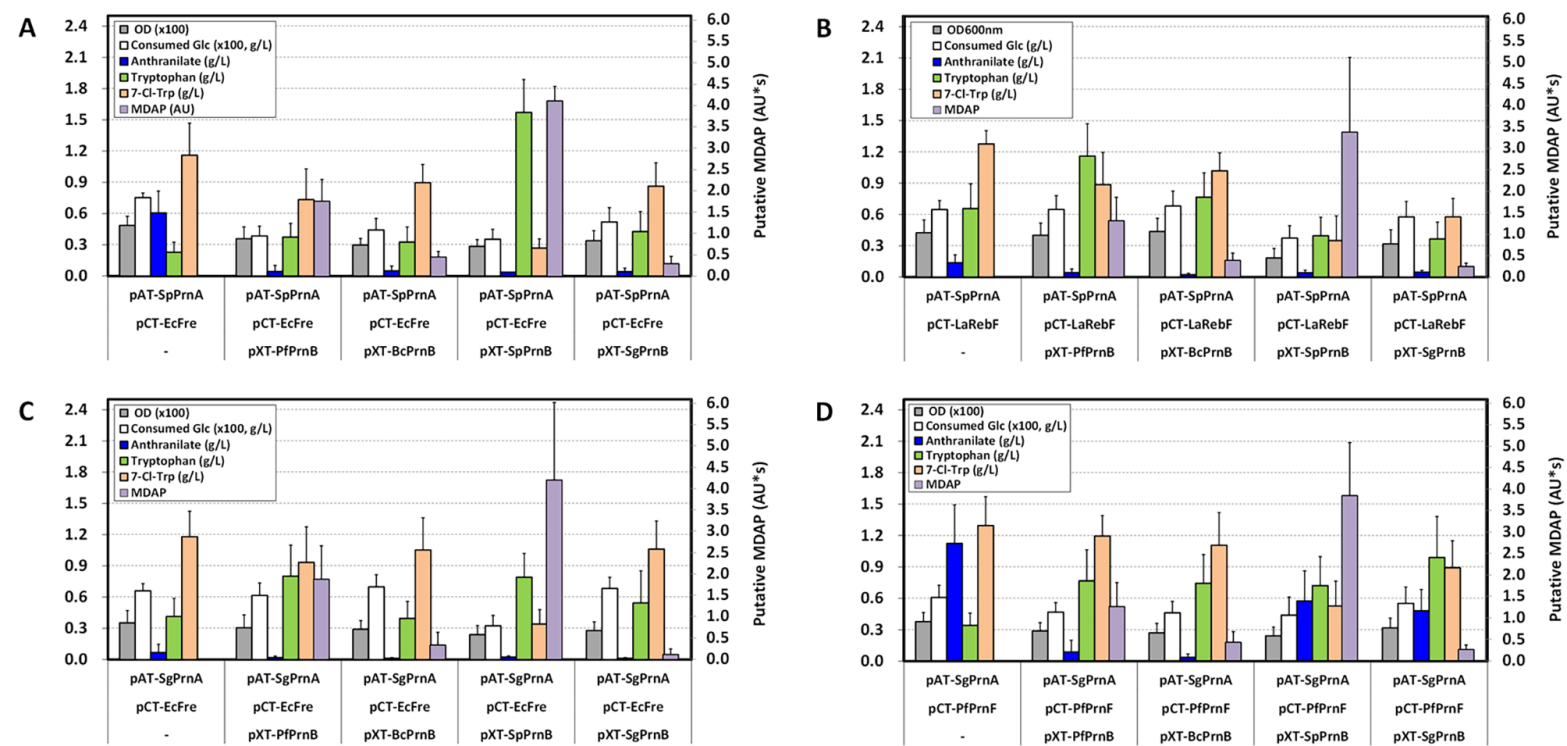
Production of putative monodechloroaminopyrrolnitrin (MDAP) and its precursors by recombinant *C. glutamicum* expressing different sets of genes encoding tryptophan 7-halogenase, flavin reductase, and MDAP synthase in flask cultures. (**A**) Expression of four different MDAP synthase genes in TP851 harboring both *prnA* from *S. plymuthica* and *fre* from *E. coli*; (**B**) expression of four different MDAP synthase genes in TP851 harboring both *prnA* from *S. plymuthica* and *rebF* from *L. aerocolonigenes*; (**C**) expression of four different MDAP synthase genes in TP851 harboring both *prnA* from *S. grimesii* and *fre* from *E. coli*; (**D**) expression of four different MDAP synthase genes in TP851 harboring both *prnA* from *S. grimesii* and *prnF* from *P. fluorescens*. Cells were grown in a flask fermentation medium supplemented with 50 mM CaCl_2_ for 60 h at 32 °C with vigorous shaking at 240 rpm. Mean and standard deviation of triplicate cultivation are given

**Fig. 5:**
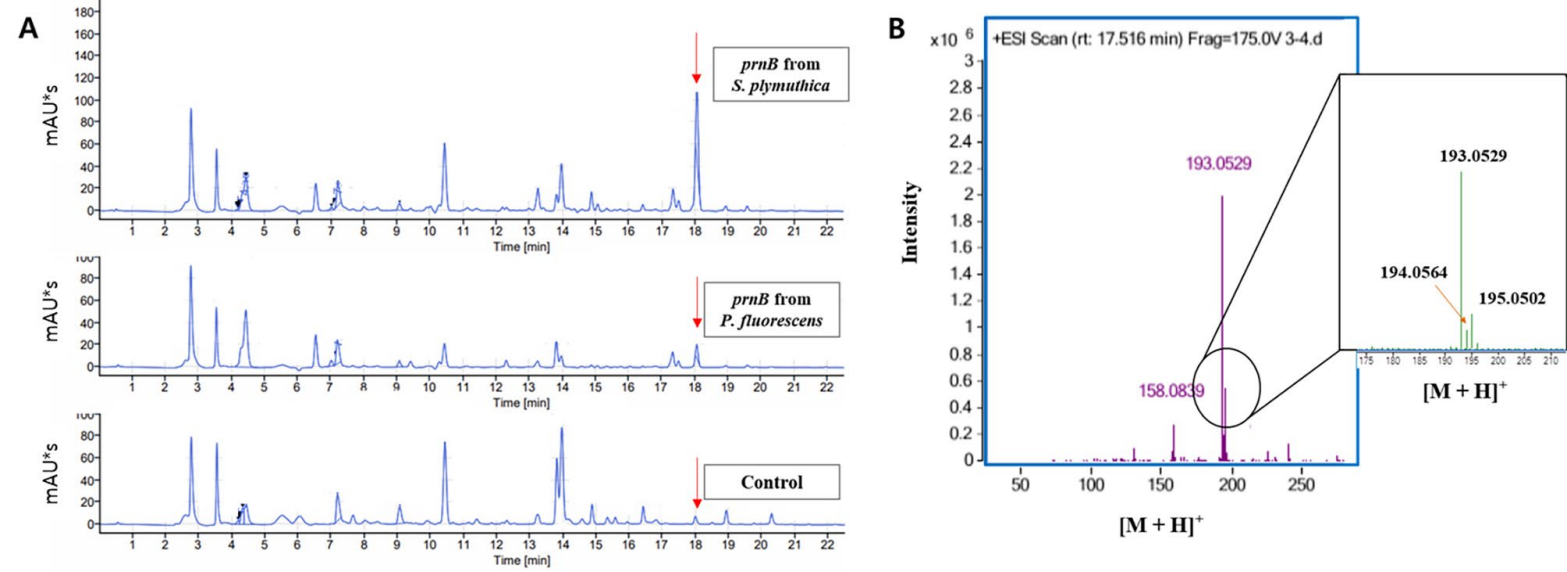
Comparative analysis of HPLC chromatograms (**A**) and mass analysis of putative monodechloroaminopyrrolnitrin by LC-QTOF-MS (**B**)

To install the MDAP biosynthetic pathway starting from l-tryptophan in *C. glutamicum*, recombinant plasmid expressing one of several *prnB*s was transformed into TP851 harboring two plasmids expressing *Sp-prnA*/*Ec-fre*, *Sp-prnA*/*La-rebF*, *Sg-prnA*/*Ec-fre*, or *Sg-prnA*/*Pf-prnF*. The constructed strains were cultured in flask fermentation medium supplemented with 50 mM CaCl_2_ for 60 h. Compared to the control TP851 harboring individual subsets of two plasmids, the expression of *Sp-prnB* in all tested TP851 harboring two plasmids resulted in not only a considerably reduced accumulation of 7-Cl-Trp but also seriously impeded both cell growth and glucose consumption (Fig. 4). Since MDAP was not available for purchase, we could not quantitatively analyze MDAP using HPLC. Nonetheless, a comparative analysis of HPLC chromatograms among the 7-Cl-Trp-producing control strain and several *prnB*-expressing strains revealed a new peak with a retention time of 18.1 min in TP851 expressing both *Sp-prnB* or *Pf-prnB* and two-component halogenase/reductase system, but not in TP851 expressing only two-component halogenase/reductase system (Fig. 5A). After collecting this peak fraction, its molecular mass was determined using Q-TOF mass spectrometry. In positive mode, three peaks representing [M + H]^+^ of 193.0529, 194.0564, and 195.0502 were detected, consistent with the theoretical monoisotopic MDAP mass of 192.0454 (Fig. 5B). As a result, the putative MDAP productions in all evaluated TP851 strains with a two-component halogenase/reductase system were the highest in cells expressing *Sp-prnB*, followed by *Pf-prnB*. In particular, the introduction of *Sp-prnB* in TP851 with expression of both *Sg-prnA* and *Ec-fre* (named MAP463) led to the highest MDAP absorbance units*Sect. (4.2 AU*s) among the TP851 strains tested (Fig. 4C). Combinatorial expression of *Sp-prnA*/*Ec-fre*/*Sp-prnB* (named MAP363) and *Sp-prnA*/*Pf-prnF*/*Sp-prnB* (named MAP383), respectively, showed 4.11 AU*s and 3.85 AU*s of putative MDAP. However, as a consequence of Sp-PrnB introduction in the strain MAP463, cell growth and consumed glucose concentrations were 68% and 49%, respectively, compared to the control strain, suggesting that the decreased cell growth and delayed glucose consumption resulted from cytotoxicity of accumulated MDAP against *C. glutamicum*. Given analyses of SDS-PAGE and putative MDAP production, we revealed that the PrnB from *S. plymuthica* among several introduced *prnB*s is able to convert 7-Cl-Trp to MDAP most efficiently, thereby reducing 7-Cl-Trp concentrations and increasing putative MDAP titers. Furthermore, since Ec-Fre showed the best performance in 7-Cl-Trp production, followed by Pf-PrnF and LaLebF (Fig. 3), in the next study evaluating APRN production using engineered *C. glutamicum*, three sets, namely Sp-PrnA/Sp-PrnB/Ec-Fre, Sg-PrnA/Sp-PrnB/Ec-Fre, and Sg-PrnA/Sp-PrnB/Pf-PrnF, were selected (Fig. S8).

**Fig. 6:**
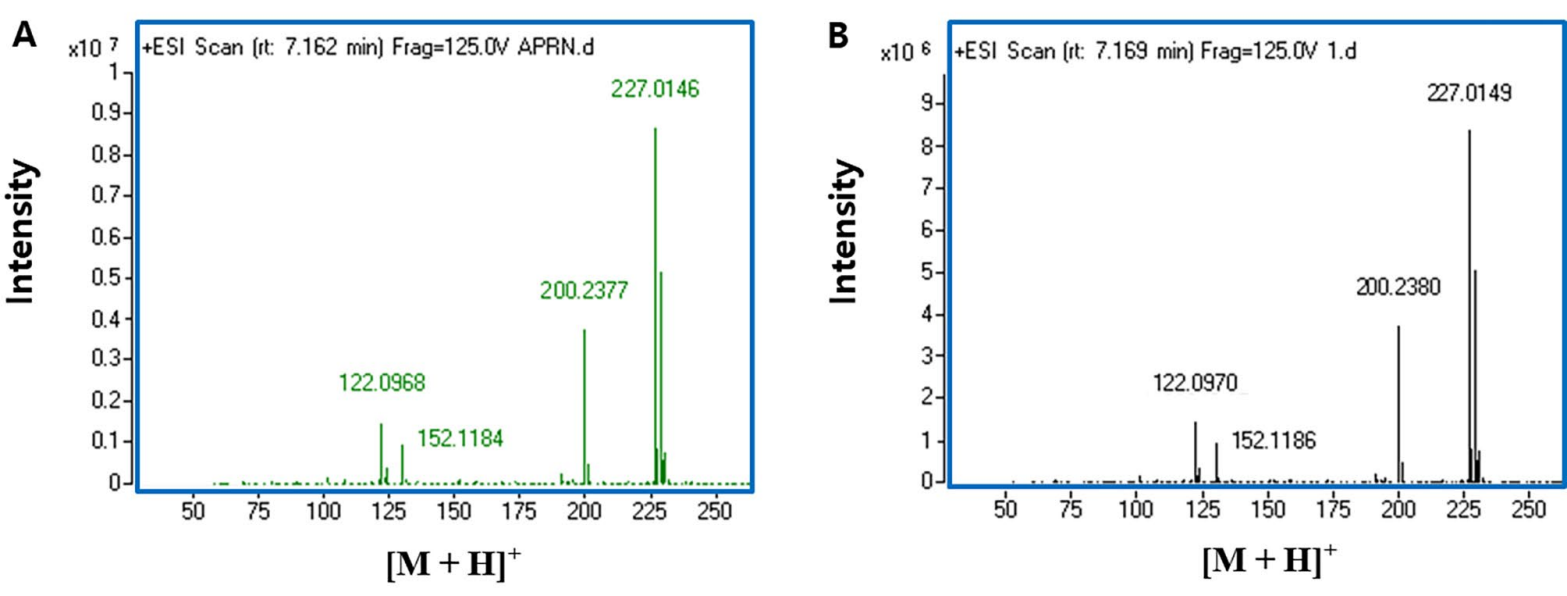
Identification of aminopyrrolnitrin isolated from fermentation broth by LC-QTOF-MS analysis. (**A**) Synthetic aminopyrrolnitrin; (**B**) aminopyrrolnitrin isolated from fermentation broth

To assemble the APRN biosynthetic pathway starting from l-tryptophan, four *prnC*s were subcloned into pCT-EcFre and pCT-PfPrnF. The resulting derivatives were then transformed into TP851 containing pAT-SgPrnA and pXT-SpPrnB. The pCT-EcFre derivatives each harboring four *prnC*s were transformed into TP851 with pAT-SpPrnA and pXT-SpPrnB. Of these assembled strains, when *Sg-prnC* and *Sp-prnC*, respectively, were introduced into MAP363 which harbored plasmid-borne expressions of *Sp-prnA*, *Ec-fre*, and *Sp-prnB*, APN3639 and APN3638 accumulated 29.5 mg/L and 28.1 mg/L of APRN in the culture broth after 60 h (Table 2). The recombinant TP851 expressing *Sg-prnA, Pf-prnF, Sp-prnB*, and *Sp-prnC* (named APN4838) produced 26.4 mg/L of APRN. However, introduction of *Pf-prnC* or *Bc-prnC* into the engineered strains disabled production of APRN in flask cultures. On the one hand, to confirm whether the APRN produced fermentatively by the engineered APN3639 strain was an authentic APRN, the APRN isolated from flask broth was analyzed by Q-TOF mass spectrometry. Bio-APRN isolated from fermentation culture and synthetic APRN had the same molecular mass [M + H]^+^ = 227.015 and splitting patterns, as shown in Fig. 6. Accordingly, we illustrated that a bio-APRN is an authentic APRN. Taken together, APRN production was successfully achieved through combinatorial expression of *prnA* from *S. plymuthica* or *S. grimesii*, *prnB* from *S. plymuthica*, and *prnC* from *S. plymuthica* or *S. grimesii*, together with expression of flavin reductase gene, *Ec-fre* or *Pf-prnF*, in engineered *C. glutamicum* (Fig. S8).

**Table 2:** Production of aminopyrrolnitrin and its precursors by recombinant *C. glutamicum* TP851 harboring genes related to artificial aminopyrrolnitrin biosynthetic pathway

Strain/plasmid 1	Plasmid 2	Plasmid 3	OD_600nm_	Consumed glucose (g/L)	ANT^*^ (g/L)	TRP^**^ (g/L)	7-Cl-Trp^***^ (g/L)	Putative MDAP (AU*s)^†^	APRN^††^ (mg/L)
TP851/pAT-SpPrnA	pXT-SpPrnB	-	21.8 ± 7.6	23.5 ± 8.5	0. 10 ± 0.14	0.62 ± 0.08	0.43 ± 0.19	2.95 ± 0.75	0
		pCT-EcFre-PfPrnC	25.6 ± 9.3	24.9 ± 9.1	< 0.05	0.98 ± 0.08	0.52 ± 0.17	3.57 ± 1.71	0
		pCT-EcFre-BcPrnC	34.0 ± 8.6	36.3 ± 10.4	< 0.05	1.10 ± 0.24	0.66 ± 0.09	4.51 ± 1.33	0
		pCT-EcFre-SpPrnC	26.3 ± 9.1	28.2 ± 7.6	< 0.05	1.08 ± 0.35	0.41 ± 0.06	0.10 ± 0.13	28.1 ± 6.4
		pCT-EcFre-SgPrnC	26.8 ± 7.5	30.5 ± 11.6	< 0.05	0.43 ± 0.14	0.56 ± 0.14	0.06 ± 0.08	29.5 ± 20.3
TP851/pAT-SgPrnA	pXT-SpPrnB	-	30.3 ± 3.4	41.7 ± 7.3	< 0.05	0.56 ± 0.26	0.82 ± 0.14	5.38 ± 1.31	0
		pCT-EcFre-PfPrnC	29.8 ± 9.7	41.9 ± 14.0	< 0.05	0.36 ± 0.21	0.47 ± 0.27	3.41 ± 1.59	0
		pCT-EcFre-BcPrnC	32.3 ± 10.2	50.3 ± 14.4	0.13 ± 0.07	< 0.05	0.07 ± 0.03	1.29 ± 0.38	0
		pCT-EcFre-SpPrnC	14.8 ± 5.6	18.3 ± 2.7	< 0.05	0.11 ± 0.14	0.19 ± 0.05	0	15.2 ± 11.1
		pCT-EcFre-SgPrnC	7.9 ± 2.3	22.4 ± 2.8	< 0.05	0.42 ± 0.28	0.23 ± 0.06	0.24 ± 0.36	14.9 ± 3.2
TP851/pAT-SgPrnA	pXT-SpPrnB		33.8 ± 8.4	40.9 ± 4.0	0.64 ± 0.41	1.81 ± 0.14	0.27 ± 0.12	3.73 ± 1.30	0
		pCT-PfPrnF-PfPrnC	46.3 ± 4.4	74.0 ± 5.2	0.29 ± 0.25	0.77 ± 0.28	0.90 ± 0.22	6.40 ± 2.78	0
		pCT-PfPrnF-BcPrnC	37.6 ± 8.3	76.5 ± 7.5	< 0.05	0.36 ± 0.25	0.64 ± 0.18	0.15 ± 0.12	0
		pCT-PfPrnF-SpPrnC	21.5 ± 9.2	26.0 ± 16.9	< 0.05	0.34 ± 0.29	0.48 ± 0.17	0	26.4 ± 5.0
		pCT-PfPrnF-SgPrnC	15.7 ± 3.2	20.9 ± 4.8	< 0.05	0.63 ± 0.26	0.27 ± 0.19	1.43 ± 0.71	21.7 ± 13.3

ANT^*^, anthranilate; TRP^**^, l-tryptophan; 7-Cl-Trp^***^, 7-chloro-l-tryptophan; MDAP (AU)^†^, monodechloroaminopyrrolnitrin (absorbance units*sec); APRN^††^, aminopyrrolnitrin. Cells were grown in a flask fermentation medium supplemented with 50 mM CaCl_2_ for 60 h at 32 °C with vigorous shaking at 240 rpm. Mean and standard deviation of triplicate cultivation are given

## Discussion

De-regulation of feedback inhibition of key enzymes catalyzing committed steps is one of the basic strategies in strain engineering for amino acids production [38, 60]. Tryptophan biosynthesis in *C. glutamicum* is mainly regulated by feedback inhibition of anthranilate synthase (TrpE) and anthranilate phosphoribosyltransferase (TrpD) by the end-product l-tryptophan [18]. As seen in *C. glutamicum* TP679, TrpD activity was inhibited by l-tryptophan, resulting in the accumulation of anthranilate (Table 1; Fig. 2). O’gara and Dunican identified the feedback-resistant TrpD mutation (S149F and A162E) conferring 80% 5-methyltryptophan resistance in *C. glutamicum* [40]. When we introduced various TrpD mutants at residue 162, all four mutants alleviated the feedback inhibition of TrpD by l-tryptophan, proving that the A162 residue is pivotal for allosteric inhibition of TrpD by l-tryptophan (Fig. 2A). On the bases of degrees of both resistance to feedback inhibition and residual activity in the presence of l-tryptophan as well as substrate affinity and assessment of l-tryptophan and anthranilate production, the A162D represented the most promising mutant for engineering of l-tryptophan-producing strain. However, both TP679 harboring A162S or A162T in plasmid and TP851 with two copies of *trpD*^A162D^ in the genome still accumulated high titers of anthranilate, which may be attributed to the fact that many sources of TrpDs are tightly regulated by the substrate anthranilate [49]. Indeed, A162D was strongly inhibited by anthranilate above 1.5 µM (data not shown). Therefore, detailed kinetic studies, including inhibition constant by anthranilate for the wild-type and mutant TrpDs, are needed to determine the causality of anthranilate and l-tryptophan production by employed mutant TrpDs in TP679.

As a green alternative to more hazardous chemical routes, regioselective halogenation of l-tryptophan by enzymes has increasingly attracted for biocatalytic C-H functionalization [48]. 7-Halogenation of l-tryptophan was successfully implemented using RebH and RebF in l-tryptophan-producing *C. glutamicum*, resulting in accumulation of 108 mg/L of 7-Cl-Trp or 0.49 g/L of 7-Br-Trp in flask cultures [56, 57]. In an attempt to further improve halogenation ability, we evaluated two-component flavin-dependent halogenase/reductase systems from various sources in *C. glutamicum*. The combined expression of *prnA* from *S. grimesii* or *S. plymuthica* with flavin reductase gene from *E. coli*, *P. fluorescens*, or *L. aerocolonigenes* yielded higher production of 7-Cl-Trp in comparison to other sets of two-component systems. Notably, PrnA from *S. grimesii* and Fre from *E. coli* in the CT46 strain represented the best two-component monooxygenase system and enabled production of 1.12 g/L of 7-Cl-Trp, indicating the highest level reported to date. Consequenly, PrnA from *S. grimesii* or *S. plymuthica* assembled with Fre from *E. coli* is believed to provide more robust halogenation capability than the RebH/RebF system. Despite the robust in vivo activity of Sg-PrnA/Ec-Fre system, 0.44 g/L of l-tryptophan remained in the culture broth of the strain CT46. One of hurdles to improving halogenation efficiency is the inefficient coupling of FADH_2_ between reductase and halogenase, where diffusing FADH_2_ could be intercepted by O_2_ in the medium to form hydrogen peroxide as a byproduct, and thus about 230 molecules of FADH_2_ will be required for a single chlorination reaction [1, 62]. Direct transfer of FADH_2_ from flavin reductase to monooxygenase through protein-protein interaction would prevent the flavin from being oxidized. Typical examples of protein-protein interaction in the two-component system have been identified between *E. coli* SsuE and SsuD, *Vibrio harveyi* NADPH-FMN oxidoreductase and luciferase, and *P. fluorescens* Pf-5 PrnF and PrnD [1, 31, 32]. Compared to other two-component monooxygenase systems in APRN biosynthesis, there were no reports of direct transfer of FADH_2_ to PrnAs, RebH, or PrnCs, which suggest that FADH_2_ generated by Fre, RebF, and Pf-PrnF is transferred to halogenases through passive diffusion [9, 11, 62]. To address halogenation efficiency, Andorfer et al. [2] reported that RebH-RebF fusion protein allowed to an increase of 7-Cl-Trp by 2.5-fold compared to RebH + RebF through inducing a high local concentration of FADH_2_ and increasing solubility of proteins. From this perspective, protein fusion in a two-component system is considered one of the feasible approaches to enhancing 7-Cl-Trp production in vivo and in vitro.

When engineered strains producing 7-Cl-Trp, MDAP, or APRN were cultured in flask fermentation medium, cell growth and d-glucose consumption were gradually retarded in accordance with accumulation of metabolites (Figs. 3 and 4; Table 2). The half-maximal growth rates of *C. glutamicum* ATCC 13,032 were observed at concentrations of 0.1 mM (24 mg/L) 7-Cl-Trp and 0.32 mM (91 mg/L) 7-Br-Trp, respectively [56, 57]. TrpD activity was reduced to about one-third by 0.05 mM 7-Cl-Trp or 0.15 mM 7-Br-Trp, implying that the growth inhibition of *C. glutamicum* may be partially due to TrpD inhibition by halogenated tryptophans [56]. Even though the growth of *C. glutamicum* ATCC 13,032 was highly sensitive to 7-Cl-Trp, the strain CT46 was able to produce up to 1.12 g/L of 7-Cl-Trp. This is presumed to be because TrpD of the strain CT46 exhibits feedback resistance to l-tryptophan, so it responds relatively less sensitively to the accumulation of 7-Cl-Trp compared to the wild-type strain. On the one hand, the perturbation of cell growth and d-glucose consumption by MDAP and APRN was more serious than that by 7-Cl-Trp in *C. glutamicum*. In particular, production titer of APRN was very low compared to that of 7-Cl-Trp. Up to now, the toxicity of MDAP and APRN to microorganisms is unknown. PRN strongly inhibits the growth of fungi and, to a lesser extent, yeast and Gram-positive bacteria such as *Agrobacterium tumefaciens, M. tuberculosis*, and *Corynebacterium insidiosum* [42]. Primary damage caused by PRN occurred in the cell membrane of *Bacillus megaterium* by combining with some phospholipids [39]. In terms of chemical structure, MDAP and APRN are chlorinated phenylpyrrole derivatives similar to PRN [19]. Thus, it is assumed that production of MDAP and/or APRN in *C. glutamicum* likely affects the cell membrane through a similar mechanism of action as PRN.

PRN, which harbors a strong antimicrobial activity, is one of the representative natural halogenated phenylpyrrole derivatives, and PRN-producing strains have been isolated from many bacteria, including *P. pyrrocinia*, *P. fluorescens*, *P. aureofaciens*, *S. marcescens*, *S. plymuthica*, *B. cepacia*, etc [19, 42]. . Recently, it was confirmed that APRN also exhibits significant antifungal activity similar to PRN [24]. In this study, an artificial APRN biosynthetic pathway was installed in *C. glutamicum* and resulted in production of mg-scale APRN in flask culture for the first time. Contrary to expectations, APRN titers were at very low level, possibly due to toxicity of both APRN and its precursors to the host strains. Indeed, PRN production of isolated bacteria was also less than 100 mg/L depending on media composition [42, 45]. As well, metabolically engineered strains from heterologous bacteria to overproduce PRN have not been known. Hence, microbial production of mg-scale APRN is considered a successful achievement and will serve as a starting point for g-scale production of APRN in *C. glutamicum*. In the following studies, plausible approaches to address the challenges in strain and process development for APRN overproduction may include: alleviating cytotoxicity of 7-Cl-Trp, MDAP, and/or APRN based on adaptive laboratory evolution, optimizing FADH_2_ regeneration efficiency, enhancing catalytic efficiency of corresponding key enzymes, or adopting bioconversion process from l-tryptophan to APRN [2, 44, 50].

Since PRN is easily decomposed by light due to poor photostability, its application has limited except for the treatment of superficial dermatophytic fungal infections [42, 46]. Alternatively, synthetic fludioxonil, a structurally similar chemical with increased photostability in comparison to PRN and a high antifungal activity, is currently commercially available as a synthetic non-systemic surface fungicide for seed and foliar treatment of plants [21]. With long-term use of fludioxonil, it has been reported to cause a high toxicity to aquatic organisms, acts as a potential endocrine disruptor in human cell lines, and gives rise to appearance of resistant field strains [4, 12, 17, 33, 54, 58, 63]. Taking into account food and environmental health risks, the European Food Safety Authority has established maximum residue levels for fludioxonil in various crops, vegetables, and fruits [10]. In this work, we demonstrated that APRN displays higher photostability than PRN. Therefore, APRN seems to be regarded as having high potential to be developed as a novel alternative biofungicide that offers high photostability, strong antifungal activity, low toxicity, and eco-friendliness.

## Conclusion

APRN production in *C. glutamicum* was achieved through engineering of anthranilate phosphoribosyltransferase along with the expression of genes encoding tryptophan 7-halogenase, flavin reductase, MDAP synthase, and MDAP halogenase from serveral microbial sources. As a result, the APN3639 accumulated 29.5 mg/L of APRN in culture broth of baffled flasks. This marks the first report on the fermentative APRN production by metabolically engineered *C. glutamicum*.

## Materials and methods

### Bacterial strains, plasmids, and general genetic manipulations

All bacterial strains and plasmids used are listed in Table S1 and S2. *E. coli* Top10 was used as a host strain for plasmid construction. *C. glutamicum* TP679 and TP851, respectively, were employed as mother strains for constructing l-tryptophan- and APRN-producing strains [57] (Table S1). Plasmids pCXE50, pCEX50T, pCXS35, and pCXI43 were used for gene expression and the shuttle vectors pCES208 and pALT601 were used for subcloning of gene inwere used for subcloning of gene in *C. glutamicum* [27, 41, 53] (Table S1). DNA fragments obtained *via* PCR (polymerase chain reaction) in all constructed plasmids were confirmed by DNA sequencing.

### Culture media and conditions

*E. coli* and *C. glutamicum* were routinely grown in lysogeny broth (LB) medium at 150 rpm at 37 °C and 32 °C, respectively. When necessary, 10 mg/L l-phenylalanine, 10 mg/L l-tyrosine, 50 mg/L spectinomycin, 50 mg/L kanamycin, 25 mg/L chloramphenicol for *E. coli*, and/or 4.8 mg/L chloramphenicol for *C. glutamicum* were added to LB medium. For metabolite production, *C. glutamicum* was inoculated into 25 mL of fermentation production medium supplemented with 250 mg/L of l-phenylalanine and l-tyrosine in 250 mL baffled flasks at 240 rpm for 48–60 h [53]. Fermentation process was conducted three times and the means and errors from triplicates were calculated. To produce 7-Cl-Trp, MDAP, and APRN 50 mM CaCl_2_ were added to flask fermentation medium. Cell growth was monitered by measuring the optical density at 600 nm using a UV-2550 spectrophotometer (Shimadzu, Japan).

### Expression vector construction for *C. glutamicum*

Expression vector pCXE50T with a truncated transcription terminator (T_*rrnB*s_) was constructed by ligating 5 kb pYL250/*Not*I/*Hin*dIII with 0.344 kb P_*tuf*_/*Not*I/*Hin*dIII from pCXE50 [22] (Table S1, Fig. S1A). Another *E. coli*/*C. glutamicum* expression vector pALT601 with a different replication origin and spectinomycin marker was constructed by assembling 4.19 kb fragment of pAL-rfp_T/*Ssp*I/*Eco*RI with 0.625 kb amplified fragment of P_*tuf*_-T_*rrnB*s_*via* PCR using primers Tuf-F and Trrn-R and pCXE50T as a template DNA [6] (Table S1, S3, and Fig. S1B).

### Plasmid construction for anthranilate phosphoribosyltransferase mutants from *C. glutamicum*

A 1.05 kb fragment of wild-type *trpD* from *C. glutamicum* was amplified *via* PCR using primer set trpD-F and trpD-R and cloned into pCXE50/*Eco*RI/*Hin*dIII to generate pX-A162, and then subcloned into pCES208 to obtain pC-A162 (Table S1). To introduce mutations in which Ala162 (A162) residue of TrpD is substituted with Asp (A162D, GCG → GAT), Glu (A162E, GCG → GAG), Ser (A162S, GCG → TCG), and Thr (A162T, GCG → ACG), we amplified 5’- and 3’-regions of *trpD* gene *via* PCR using primer sets trpD-F/trpD-D-R and trpD-D-F/trpD-R, trpD-F/trpD-E-R and trpD-E-F/trpD-R, trpD-F/trpD-S-R and trpD-S-F/trpD-R, and trpD-F/trpD-T-R and trpD-T-F/trpD-R, respectively (Table S3). The amplified fragments of both regions were assembled with pCXE50/*Eco*RI/*Hin*dIII *via* the Gibson Assembly to produce pX-A162D, pX-A162E, pX-A162S, and pX-A162T, respectively, and then sublconed into another shuttle vector pCES208, yielding pC-A162D, pC-A162E, pC-A162S, and pC-A162T [13] (Table S1).

### Strain construction for tryptophan-producing *C. glutamicum*

Plasmid pK19-*trpD*^A162D^ was obtained by assembling pK19*mobsacB*/*Hind*III/*Eco*RI [47] with amplified 0.6 kb partial fragment of *trpD*^A162D^ using primers trpD-D-sF and trpD-D-sR (Table S1 and S3). To introduce *trpD*^A162D^ mutation, pK19-*trpD*^A162D^ was transformed into TP679 and followed by two-step homologous recombination. The point mutation in a resulting TP793 was confirmed through PCR using primers trpD-con-F and trpD-con-R and DNA sequencing (Fig. S2A and Table S3). To insert *trpD*^A162D^ into the *NCgl1112*-deleted locus (encoding maleylacetate reductase) in the genome, plasmid pK19-*Δ1112* was obtained by assembling pK19*mobsacB*/*Hind*III/*Eco*RI with amplified 1 kb partial fragments of *NCgl1111* and *NCgl1113* using primer sets 1111-F/1111-R and 1113-F/1113-R, respectively (Table S1 and S3). Plasmid pK19-*Δ1112*::P_*tuf*_*-trpD*^A162D^ was constructed by ligating pK19-*Δ1112*/*Not*I/*Xba*I with 1.8 kb P_*tuf*_-*trpD*^A162D^-T_*rrnB*_ fragment obtained from *Not*I/*Nhe*I-treated pX-A162D plasmid. pK19-*Δ1112*::P_*tuf*_*-trpD*^A162D^ was transformed into TP793, yielding TP851 (Table S1). The insertion of an additional copy of *trpD*^A162D^ into the partially deleted *NCgl1112* locus (0.48 kb) encoding was confirmed by PCR analysis using primers C-1111-F and C-1113-R (Fig. S2B, Table S3).

### Plasmid construction for expression of genes in the APRN biosynthetic pathway

All genes with optimized *C. glutamicum* codon usage except *fre* encoding flavin reductase from *E. coli* were synthesized by Integrated DNA Technologies (IDT, Iowa, U. S. A.). Plasmid pX-PfPrnA was constructed by assembling codon-optimized *prnA* from *P. fluorescens* (*Pf-prnA*) with pCXE50/*Eco*RI/*Hin*dIII (Table S2 and S4). The cloned *Pf-prnA* was subcloned into pCXS35 and pCXI43 to generate pX35-PfPrnA and pX43-PfPrnA, respectively. Plasmid pX-LaRebH was obtained by amplifying 1.58 kb *rebH* from *L. aerocolonigenes* using synthesized *rebHF* DNA fragment as a template and primer set rebH-F and rebH-R and then cloned in pCXE50/*Eco*RI/*Hin*dIII (Table S3 and S5). The cloned *rebH* gene in pX-LaRebH was sublcloned into the expression vector pCXE50T/*Eco*RI/*Hin*dIII to generate pXT-LaRebH. Codon-optimized genes encoding tryptophan 7-halogenase from *S. plymuthica* and *S. grimesii* were cloned *via* the Gibson Assembly in the pCXE50T digested with *Eco*RI and *Hin*dIII (Table S6 and S7). The constructed plasmids designated pXT-SpPrnA and pXT-SgPrnA, respectively (Table S2). The cloned genes in pX43-PfPrnA, pXT-SpPrnA, pXT-SgPrnA, and pX-LaRebH were subcloned into another shuttle vecotr pALT601 to yield pA43-PfPrnA, pAT-SpPrnA, pAT-SgPrnA, and pA-LaRebH, respectively (Table S2). Codon-optimized genes coding for (hypothetical) flavin reductases from *P. fluorescens*, *S. plymuthica*, and *S. grimesii* were cloned in the pCXE50T/*Eco*RI/*Hin*dIII to obtain pXT-PfPrnF, pXT-SpPrnF, and pXT-SgPrnF, respectively (Table S2, S8, S9 and S10). The 0.51 kb *rebF* fragment encoding flavin reductase from *L. aerocolonigenes* was obtained *via* PCR from synthesized *rebHF* DNA using primer set rebF-F and rebF-R and cloned into pCXE50T/*Eco*RI/*Hin*dIII to make pXT-LaRebF (Table S2, S3, and S5). The *fre* gene from *E. coli* was amplified *via* PCR using primer set fre-F and fre-R and *E. coli* genomic DNA as a template, and then assembled with *Eco*RI- and *Hin*dIII-treated pCXE50T, resulting in plasmid pXT-EcFre. The cloned genes described above were subcloned into another shuttle vecotr pCES208 to yield pCT-PfPrnF, pCT-SpPrnF, pCT-SgPrnF, pCT-LaRebF, and pCT-EcFre (Table S2). The codon-optimized 1.086 kb *prnB*s from *P. fluorescens* BL915, *S. plymuthica* PR1-2 C, and *S. grimesii* as well as 1.083 kb *prnB* from *Burkholderia contamins* were synthesized from IDT (Table S11, S12, S13 and S14). The codon-optimized 1.704 kb *prnC*s from *P. fluorescens* BL915, *S. plymuthica* PR1-2 C, and *S. grimesii* as well as 1.698 kb *prnC* from *B. contamins* were synthesized from IDT (Table S15, S16, S17 and S18). The synthesized DNAs flanking with both upstream 20 nucleotides (*Eco*RI site) and downstream 20 nucleotides (*Hin*dIII site) were fused to pCXE50T/*Eco*RI/*Hin*dIII *via* the Gibson assembly method, generating the construction of pXT-PfPrnB, pXT-SpPrnB, pXT-SgPrnB, pXT-BcPrnB, pXT-PfPrnC, pXT-BcPrnC, pXT-SpPrnC, and pXT-SgPrnC, respectively (Table S2). The cloned *prnC* genes digested with *Not*I and *Nhe*I were sublconed into *Not*I and *Xba*I-treated pCT-EcFre and pCT-PfPrnF, respectively, resulting in construction of pCT-EcFre-PfPrnC, pCT-EcFre-BcPrnC, pCT-EcFre-SpPrnC, pCT-EcFre-SgPrnC, pCT-PfPrnF-PfPrnC, pCT-PfPrnF-BcPrnC, pCT-PfPrnF-SpPrnC, and pCT- PfPrnF-SgPrnC (Table S2).

### Protein assay and SDS-PAGE

Overnight cultured cells in 10 mL LB medium supplemented with antibiotic at 32 °C were harvested by centrifugation at 5,000 rpm for 5 min and washed with phosphate-buffered saline (PBS) twice. Collected cells in 0.5 mL PBS buffer were disrupted by using microbeads with vigorous vortexing for 5 min (3–5 times), followed by centrifugation at 13,000 rpm for 15 min. Protein concentration was determined by BCA protein assay kit (Thermo Fisher Scientific, U.S.A.) with BSA as a standard. 10 µg of proteins dissolved in loading buffer were heated for 5 min at 85 °C and added to the well of 10% SDS-PAGE gel. After running, the gel was soaked in 2% Coomassie blue staining solution (LPS Solution, South Korea) for 1–2 h and destained with distilled water.

### Determination of anthranilate phosphoribosyltransferase TrpD activity

Recombinant TP679 strains expressing wild-type and various mutant alleles of *trpD* from an expression plasmid were inoculated from an overnight culture and was cultivated for 24 h in LB medium with 50 mg/L l-tryptophan at 30 °C with 120 rpm before cells were centrifuged for 10 min at 4 °C and 4,000 rpm and stored at -20 °C. After resuspension in 100 mM Tris-HCl buffer (pH 7.4), the cells were sonicated for 9 min at 55% amplitude and 0.5 cycles on ice in the UP200S Ultrasonic Processor from Hielscher Ultrasound Technology. The supernatant obtained after centrifugation (60 min, 4 °C, 16,400 rpm) was used as crude extract for the enzyme assay. The activity was assayed fluorometrically by monitoring the decrease of anthranilate fluorescence at room temperature. The reaction mixture with a final volume of 1 mL contained 100 mM Tris-HCl buffer (pH 7.4), 100 mM MgCl_2_, 3 µM anthranilate, 0.3 mM PRPP, and the crude extract and was filled into a quartz glass cuvette [56] (Hellma Analytics, High Precision cell, Light Path 10 × 4 mm). When necessary, 0.15, 0.3, 0.6, 1, 2, 5 mM l-tryptophan, respectively, was added in reaction mixture. Anthranilate fluorescence was detected with 325 nm excitation and 400 nm emission wavelengths using the Shimadzu Spectrofluorophotometer RF-5301PC. Protein concentrations were determined by the Bradford method [3] with bovine serum albumin as reference. Means and errors from triplicates were calculated.

### Determination of photostability of APRN and PRN

Samples were taken at intervals of 0, 10, 20, 30, and 60 min while irradiating ultraviolet light at a height of 20 cm to the APRN and PRN solutions dissolved in an aprotic acetonitrile solvent, respectively, and the concentrations were measured using HPLC.

### Chemicals

The reagents anthranilate and l-tryptophan were purchased from SIGMA-ALDRICH (U.S.A.), and 7-Cl-dl-tryptophan was purchased from Accela ChemBio Inc. (Jiangxi, China). Aminopyrrolnitrin and pyrrolnitrin were purchased from WuXi AppTec. (Wuxi, China).

### Analytic procedure of HPLC and LC-QTOF-MS

HPLC samples were prepared by mixing 100 µL of fermentation broth with 900 µL of methanol in an e-tube, centrifuging for 10 min at 13,000 rpm, and filtering using a 0.45 μm RC membrane filter (Sartorius, Germany). Quantification of anthranilate, l-tryptophan, 7-Cl-Trp, APRN, and PRN was carried out using Agilent 1260 equipped with a Poroshell 120-EC-C18 column (Agilent 4.6 × 150 mm, 2.7 μm) at a flow rate of 0.5 mL per min. For the mobile phase, 0.1% formic acid in deionized water and 0.1% formic acid in acetonitrile were used with gradient elution. 10 µL of each sample were injected into the system and eluted for 35 min for each sample analysis at 50 °C of column temperature. Anthranilate, putative MDAP, and APRN were detected at 310 nm, whereas l-tryptophan, 7-Cl-Trp, and PRN were detected at 280 nm. Sample-preparing steps for LC-QTOF-MS (Quadrupole time-of-flight mass spectrometry) analysis were the same as HPLC sample-preparing steps. First, the sample was run using Agilent 1290 Infinity equipped with Poroshell 120-SB-C18 (Agilent 2.1 × 100 mm, 2.7 μm) at 0.5 mL/min flow rate in gradient elution. Next, the mass of the sample was analyzed using Agilent 6530 QTOF with positive ionization.

### Electronic supplementary material

Below is the link to the electronic supplementary material.

Supplementary Material 1

## Data Availability

No datasets were generated or analysed during the current study.
