# Supplementary material for: Fermentative aminopyrrolnitrin production by metabolically engineered Corynebacterium glutamicum
Source: Microb Cell Fact. 2024 May 23;23:147. doi: 10.1186/s12934-024-02424-y (PMC11112847; doi:10.1186/s12934-024-02424-y)

**Supplementary Material**

**Fermentative aminopyrrolnitrin production by metabolically engineered *Corynebacterium glutamicum***

Virginia Ryandini Melati Putri^1†^, Min-Hee Jung^1†^, Ji-Young Lee^1^, Mi-Hyang Kwak^1^, Theavita Chatarina Mariyes^1^, Anastasia Kerbs^2^, Volker F. Wendisch^2^, Hee Jeong Kong^3^, Young-Ok Kim^3^ and Jin-Ho Lee^1*^

^1^Department of Food Science & Biotechnology, BB21^+^, Kyungsung University, Busan 48434, Republic of Korea

^2^Faculty of Biology and Center for Biotechnology, Bielefeld University, Germany

^3^Biotechnology Research Division, National Institute of Fisheries Science, Busan 46083, Republic of Korea

**“*Corresponding author: Jin-Ho Lee”**

^†^These authors contributed equally to this work.

Tel: +82-51-663-4716, Fax: 82-0504-238-7072

E-mail: jhlee83@ks.ac.kr

**Supplementary Material**

**Fig. S1.** Vector maps of *E. coli*/*C. glutamicum* expression plasmids pCXE50T (A) and pALT601 (B).

**Fig. S2.** Verification of the constructed mutant strains TP793 (**A**) and TP851 (**B**) by PCR analysis. (**A**) Confirmation of a point mutation of *trpD*^A162D^ in genome of TP793. PCR was conducted by using primers trpD-con-F and trpD-con-R. Lane 1 is PCR product using TP679 chromosome as a DNA, showing no PCR band; lane 2 is PCR product using the mutant TP793 chromosome as a template DNA, showing 0.406 kb PCR band. (**B**) Confirmation of a partial deletion of *NCgl1112* locus (0.48 kb) and simultaneous insertion of P*_tuf_*-*trpD*^A162D^-T*_rrnB_* (1.794 kb) in TP792. PCR was conducted by using primers C-1111-F and C-1113-R. Lane 1 is PCR product of TP793 chromosome, showing 2.914 kb PCR band; lane 2 is PCR product of mutant TP851 chromosome, showing 4.232 kb PCR band.

**Fig. S3.** Photostability of pyrrolnitrin and aminopyrrolnitrin under UV exposure.

**Fig. S4.** SDS-PAGE of crude extracts of *C. glutamicum* ATCC 13032 expressing genes encoding tryptophan 7-halogenase from diverse sources of microorganisms. M, Molecular size marker; lanes 1, 5, pCXE50 (control); 2, pX-PfPrnA; 3, pX35-PfPrnA; 4, pX43-PfPrnA; 6, pX-LaRebH; 7, pXT-LaRebH; 8, 10, pCXE50T (control); 9, pXT-SpPrnA; 11, pXT-SgPrnA. Red arrows are expected protein bands.

**Fig. S5.** SDS-PAGE of crude extracts of *C. glutamicum* ATCC 13032 expressing genes encoding (putative) flavin reductase from diverse sources of microorganisms. M, Molecular size marker; lanes 1, 6, pCXE50T (control); 2, pXT-EcFre; 3, pXT-LaRebF; 4, pXT-PfPrnF; 5, pXT-SpPrnF; 7, pXT-SgPrnF. Red arrows are expected protein bands.

**Fig. S6.** SDS-PAGE of crude extracts of *C. glutamicum* ATCC 13032 expressing genes encoding MDAP syntase from diverse sources of microorganisms. M, Molecular size marker; lanes 1, 3, 6, pCXE50T (control); 2, pXT-BcPrnB; 4, pXT-PfPrnB; 5, pXT-SpPrnB; 7, pXT-SgPrnB. Red arrow is expected protein band.

**Fig. S7.** SDS-PAGE of crude extracts of *C. glutamicum* ATCC 13032 expressing genes encoding MDAP halogenase from diverse sources of microorganisms. M, Molecular size marker; lanes 1, 4, pCXE50T (control); 2, pXT-PfPrnC; 3, pXT-BcPrnC; 5, pXT-SpPrnC; 6, pXT-SgPrnC.

Fig. S8. Overall schematic diagram of engineered *C. glutamicum* expressing diverse sources of APRN biosynthetic pathway genes.

**Table S1.** Bacterial strains and plasmids list (1) used in this study.

| Strain or  Plasmid | | Characteristics | | | Source |
| --- | --- | --- | --- | --- | --- |
| *Escherichia coli* | | | | |  |
| Top10 | | | *F^-^ mcrA ∆(mrr-hsdRMS-mcrBC) φ80lacZ∆M15 ∆lacX74 recA1 endA1 araD139 ∆(ara-leu)7679 galU galK rps(StrR) endA1 nupG* | | Invitrogen |
| *Corynebacterium glutamicum* | | | | | |
| ATCC 13032  TP679  TP793  TP851  CT28  CT36  CT37  CT46  CT48  MAP363  MAP383  MAP463  APN3638  APN3639  APN4838 | Wild type  *Δcsm ΔtrpL::*P*_ilvC_*_-M1_ *trpE*^S38R^ *Δvdh::*P*_ilvC-_aroG*^D146N^  TP679 *trpD*^A162D^  TP793 *ΔNCgl1112::*P*_tuf_-trpD*^A162D^  TP851 harboring *La-rebH* and *Pf-prnF*  TP851 harboring *Sp-prnA* and *Ec-fre*  TP851 harboring *Sp-prnA* and *La-rebF*  TP851 harboring *Sg-prnA* and *Ec-fre*  TP851 harboring *Sg-prnA* and *Pf-prnF*  TP851 harboring *Sp-prnA, Ec-fre*, and *Sp-prnB*  TP851 harboring *Sp-prnA, Pf-prnF*, and *Sp-prnB*  TP851 harboring *Sg-prnA, Ec-fre*, and *Sp-prnB*  TP851 harboring *Sp-prnA, Ec-fre*, *Sp-prnB*, and *Sp-prnC*  TP851 harboring *Sp-prnA, Ec-fre*, *Sp-prnB,* and *Sg-prnC*  TP851 harboring *Sg-prnA, Pf-prnF*, *Sp-prnB,* and *Sp-prnC* | | | ATCC  [57]  This study  This study  This study  This study  This study  This study  This study  This study  This study  This study  This study  This study  This study | |
| Plasmid  pCXE50  pCXE50T  pYL250  pCXS35  pCXI43  pCES208  pALT601  pAL-rfp_T  pX-A162  pC-A162  pX-A162D  pC-A162D  pX-A162E  pC-A162E  pX-A162S  pC-A162S  pX-A162T  pC-A162T  pK19*mobsacB*  pK19-*trpD*^A162D^  pK19-*Δ1112*  pK19-*Δ1112::* P*_tuf_-trpD*^A162D^ | *E. coli*/*C. glutamicum* expression shuttle vector; pCXM48 derivative with P*_tuf_* and T*_rrnB_*; 5.512 kb, pGA1 *ori*V_Cg_, Cm^R^  pCXE50 derivative with P*_tuf_* and 0.231 kb truncated T_t-_*_rrnB_*; *E. coli/C. glutamicum* expression shuttle vector; 5.332 kb, pGA1 *ori*VCg, Cm^R^  pCXE50 derivative with a mutated *comt* ORF and a truncated *rrnB* transcriptional terminator (T*_rrnB_*_s_)  *E. coli/C. glutamicum* expression shuttle vector with P*_sod_*_-M_; 5.512 kb, pGA1 *ori*VCg, Cm^R^  *E. coli/C. glutamicum* expression shuttle vector with P*_ilvC_*_-M1_; 5.511 kb, pGA1 *ori*VCg, Cm^R^  *E. coli*/*C. glutamicum* shuttle vector; 5.93 kb, Kan^R^  *E. coli*/*C. glutamicum* expression shuttle vector; pAL374 derivative with P*_tuf_*-T_t-_*_rrnB_*; 4.815 kb, Spec^R^  pAL374 plasmid carrying the *rfp*(T) *sg*RNA targeting the template strand of *rfp*, Spec^R^  pCXE50 with 1.047 kb *trpD* from *C. glutamicum*; 6.535 kb  pCES208 with P*_tuf_*-*trpD*-T*_rrnB_*; 7.622 kb  pCXE50 with 1.047 kb mutant *trpD*^A162D^; 6.535 kb  pCES208 with P*_tuf_*-*trpD*^A162D^-T*_rrnB_*; 7.622 kb  pCXE50 with 1.047 kb mutant *trpD*^A162E^; 6.535 kb  pCES208 with P*_tuf_*-*trpD*^A162E^-T*_rrnB_*; 7.622 kb  pCXE50 with 1.047 kb mutant *trpD*^A162S^; 6.535 kb  pCES208 with P*_tuf_*-*trpD*^A162S^-T*_rrnB_*; 7.622 kb  pCXE50 with 1.047 kb mutant *trpD*^A162T^; 6.535 kb  pCES208 with P*_tuf_*-*trpD*^A162T^-T*_rrnB_*; 7.622 kb  Vector for allelic exchange in *C. glutamicum* (pK19*oriV_E.coli_sacBlacZα*); 5.72 kb, Kan^R^  pK19*mobsacB* derivative with 0.602 kb *trpD*^A162D^ fragment; 6.279 kb  pK19*mobsacB* derivative with *NCgl1111* (1 kb) and *NCgl1113* (1 kb) fragments; 7.699 kb  pK19*mobsacB* derivative with *NCgl1111* (1 kb), P*_tuf_*-*trpD^A162D^*-T*_rrnB_*, and *NCgl1113* (1 kb) fragments; 9.485 kb | | | [53]  This study  [22]  [27]  [27]  [41]  This study  [6]  This study  This study  This study  This study  This study  This study  This study  This study  This study  This study  [47]  This study  This study  This study | |

**Table S2.** Plasmids list (2) used in this study.

| Plasmid | | Characteristics | Source | |
| --- | --- | --- | --- | --- |
| pX-PfPrnA  pX35-PfPrnA  pX43-PfPrnA  pA43-PfPrnA  pX-LaRebH  pXT-LaRebH  pA-LaRebH  pXT-SpPrnA  pAT-SpPrnA  pXT-SgPrnA  pAT-SpPrnA  pXT-EcFre  pCT-EcFre  pXT-LaRebF  pCT-LaRebF  pXT-PfPrnF  pCT-PfPrnF  pXT-SpPrnF  pCT-SpPrnF  pXT-SgPrnF  pCT-SgPrnF  pXT-BcPrnB  pXT-PfPrnB  pXT-SpPrnB  pXT-SgPrnB  pXT-BcPrnC  pCT-EcFre-BcPrnC  pCT-PfPrnF-  BcPrnC  pXT-PfPrnC  pCT-EcFre-  PfPrnC  pCT-PfPrnF-  PfPrnC  pXT-SpPrnC  pCT-EcFre-  SpPrnC  pCT-PfPrnF-  SpPrnC  pXT-SgPrnC  pCT-EcFre-  SgPrnC  pCT-PfPrnF-  SgPrnC | pCXE50 with 1.617 kb *prnA* from *Pseudomonas fluorescens* (*Pf-prnA*); 7.105 kb  pCXS35 with 1.617 kb *Pf-prnA*; 7.105 kb  pCXI43 with 1.617 kb *Pf-prnA*; 7.104 kb  pALT601 derivative with P*_ilvC_*_-M1_-*Pf*-*prnA*-T*_rrnB_*; 6.408 kb  pCXE50 with 1.593 kb *rebH* from *Lechevalieria aerocolonigenes* (*La-rebH*); 7.081 kb  pCXE50T with 1.593 kb *La-rebH*; 6.901 kb  pALT601 derivative with P*_tuf_*-*La-rebH*-T*_rrnB_*; 6.384 kb  pCXE50T with 1.611 kb *prnA* from *Serritia plymuthica* (*Sp-prnA*); 6.919 kb  pALT601 with 1.611 kb *Sp-prnA*; 6.402 kb  pCXE50T with 1.611 kb *prnA* from *S. grimesii* (*Sg-prnA*); 6.919 kb  pALT601 with 1.611 kb *Sg*-*prnA*; 6.402 kb  pCXE50T with 0.702 kb *fre* from *Escherichia coli* (*Ec-fre*); 6.01 kb  pCES208 with P*_tuf_*-*Ec-fre*-T*_rrnB_*_s_; 7.097 kb  pCXE50T with 0.513 kb *rebF* from *L. aerocolonigenes* (*La-rebF*); 5.821 kb  pCES208 with P*_tuf_*-*La-rebF*-T*_rrnB_*_s_; 6.908 kb  pCXE50T with 0.561 kb *prnF* from *P. fluorescens*; 5.869 kb  pCES208 with P*_tuf_*-*Pf-prnF*-T*_rrnB_*_s_; 6.956 kb  pCXE50T with 0.567 kb putative *prnF* from *S. plymuthica* (*Sp-prnF*); 5.875 kb  pCES208 with P*_tuf_*-*Sp-prnF*-T*_rrnB_*_s_; 6.962 kb  pCXE50T with 0.567 kb putative *prnF* from *S. grimesii* (*Sg-prnF*); 5.875 kb  pCES208 with P*_tuf_*-*Sg-prnF*-T*_rrnB_*_s_; 6.962 kb  pCXE50T with 1.086 kb *prnB* from *Burkholderia contamins*; 6.394 kb  pCXE50T with 1.086 kb *prnB* from *P. fluorescens*; 6.394 kb  pCXE50T with 1.086 kb *prnB* from *S. plymuthica*; 6.394 kb  pCXE50T with 1.083 kb *prnB* from *S. grimesii*; 6.391 kb  pCXE50T with 1.701 kb *prnC* from *B. contamins* (*Bc-prnC*); 7.009 kb  pCT-EcFre with P*_tuf_*-*Bc-prnC*-T*_rrnB_*_s_; 9.371 kb  pCT-PfPrnF with P*_tuf_*-*Bc-prnC*-T*_rrnB_*_s_; 9.23 kb  pCXE50T with 1.704 kb *prnC* from *P. fluorescens* (*Pf-prnC*); 7.012 kb  pCT-EcFre with P*_tuf_*-*Pf-prnC*-T*_rrnB_*_s_; 9.374 kb  pCT-PfPrnF with P*_tuf_*-*Pf-prnC*-T*_rrnB_*_s_; 9.233 kb  pCXE50T with 1.704 kb *prnC* from *S. plymuthica* (*Sp-prnC*); 7.012 kb  pCT-EcFre with P*_tuf_*-*Sp-prnC*-T*_rrnB_*_s_; 9.374 kb  pCT-PfPrnF with P*_tuf_*-*Sp-prnC*-T*_rrnB_*_s_; 9.233 kb  pCXE50T with 1.704 kb *prnC* from *S. grimesii* (*Sg-prnC*); 7.012 kb  pCT-EcFre with P*_tuf_*-*Sg-prnC*-T*_rrnB_*_s_; 9.374 kb  pCT-PfPrnF with P*_tuf_*-*Sg-prnC*-T*_rrnB_*_s_; 9.233 kb | | | This study  This study  This study  This study  This study  This study  This study  This study  This study  This study  This study  This study  This study  This study  This study  This study  This study  This study  This study  This study  This study  This study  This study  This study  This study  This study  This study  This study  This study  This study  This study  This study  This study  This study  This study  This study  This study |

**Table S3.** Primer list used in this study.

| Primer | Sequence (5’ → 3’) |
| --- | --- |
| Tuf-F | TCATAACGGTTCTGGCAAATGCGGCCGCTCTAGAGCGTTT |
| Trrn-R | CGCCATAACCTCACCGAATTGGCGCCAGGGTGGTGCTAG |
| 1111-F | GCTATGACCATGATTACGCCAAGCTTCGATGCCGCTCACCTCATGC |
| 1111-R | TCTAGAATTCTTATGCGGCCGCTGTGGATCCACCGCCCACAC |
| 1113-F | GCGGCCGCATAAGAATTCTAGAGCAACAGTACTGCCTTATGTTC |
| 1113-R | GTTGTAAAACGACGGCCAGTGAATTCTCGCTTCTCTGGCGCAGTCA |
| C-1111-F | CTTCTGAAGAAAGCGCGTGG |
| C-1113-R | CTGTGGGTCATCATCGCTGT |
| trpD-F | GAAGTCCAGGAGGAGAATTCATGACTTCTCCAGCAACACTGA |
| trpD-R | CCGCCAAAACAGCCAAGCTTCTAGTCATTGGAAGACTCCTTT |
| trpD-D-R | AACCGGCTGCACATGCGCAATATCAGGGTTGTACGCAGGTGCGAA |
| trpD-D-F | TTCGCACCTGCGTACAACCCTGATATTGCGCATGTGCAGCCGGTT |
| trpD-E-R | CCGGCTGCACATGCGCAATCTCAGGGTTGTACGCAGGTGCG |
| trpD-E-F | CGCACCTGCGTACAACCCTGAGATTGCGCATGTGCAGCCGG |
| trpD-S-R | CGGCTGCACATGCGCAATCGAAGGGTTGTACGCAGGTGCGAA |
| trpD-S-F | TTCGCACCTGCGTACAACCCTTCGATTGCGCATGTGCAGCCG |
| trpD-T-R | CGGCTGCACATGCGCAATCGTAGGGTTGTACGCAGGTGCGAA |
| trpD-T-F | TTCGCACCTGCGTACAACCCTACGATTGCGCATGTGCAGCCG |
| trpD-D-sF | ACCATGATTACGCCAAGCTTCCAAGGCGTTCCTCGCGGC |
| trpD-D-sR | AAACGACGGCCAGTGAATTCGTAGCGGCCAAGGCCAAGG |
| trpD-con-F | GGGTGAATACGATGACGTGC |
| trpD-con-R | CGGCTGCACATGCGCAATAT |
| rebH-F | GAAGTCCAGGAGGAGAATTCATGTCTGGCAAGATCGACAAAA |
| rebH-R | CCGCCAAAACAGCCAAGCTTTTAACGTCCGTGCTGTTGGC |
| rebF-F | GAAGTCCAGGAGGAGAATTCATGACTATTGAGTTTGACCGCC |
| rebF-R | CAGTTCCCTACTCTAAGCTTTTAGCCTTCCGGCGTCCACA |
| fre-F | GAAGTCCAGGAGGAGAATTCATGACAACCTTAAGCTGTAAAGTG |
| fre-R | CAGTTCCCTACTCTAAGCTTTCAGATAAATGCAAACGCATCGC |

**Table S4.** Codon-optimized nucleotide sequence of *prnA* from *Pseudomonas fluorescens.*

| Codon-optimized *prnA* open reading frame from *P. fluorescens* (ORF, 1.617 kb) |
| --- |
| GAAGTCCAGGAGGAgaattc**ATG**AATAAACCGATCAAAAACATTGTTATTGTCGGTGGAGGAACTGCGGGTTGGATGGCAGCTTCGTACTTGGTGCGCGCACTTCAACAGCAAGCCAACATCACTCTCATCGAATCGGCCGCCATCCCCCGGATTGGAGTGGGTGAAGCGACTATCCCGTCGTTGCAAAAAGTGTTTTTCGACTTTTTGGGCATCCCGGAACGTGAATGGATGCCACAGGTCAACGGAGCGTTCAAGGCAGCGATCAAGTTCGTGAACTGGCGCAAGTCACCGGACCCGTCGCGGGACGACCATTTTTATCATCTTTTCGGCAATGTGCCTAACTGCGACGGAGTTCCCCTTACCCACTACTGGTTGCGCAAACGCGAACAAGGTTTTCAACAGCCTATGGAATATGCTTGCTACCCTCAGCCAGGCGCGTTGGACGGAAAATTGGCGCCATGTTTGTCGGATGGAACCCGTCAGATGTCTCACGCCTGGCATTTCGATGCGCATTTGGTCGCCGATTTTTTGAAGCGTTGGGCAGTAGAACGTGGAGTTAATCGGGTTGTCGATGAAGTAGTAGACGTGCGCTTGAACAATCGTGGATACATTTCCAACCTTCTCACGAAGGAAGGCCGTACCCTCGAGGCGGACCTTTTCATCGACTGCTCAGGTATGCGGGGTTTGCTGATTAATCAAGCCCTCAAGGAGCCGTTTATTGACATGTCGGATTACCTGTTGTGTGATTCTGCAGTCGCATCTGCCGTTCCTAATGATGACGCTCGCGACGGTGTTGAACCTTATACTTCCTCGATCGCTATGAACTCGGGATGGACCTGGAAAATTCCCATGCTGGGCCGGTTTGGATCGGGATACGTGTTTAGCTCACATTTCACGTCTCGGGACCAGGCTACTGCCGACTTCTTGAAACTTTGGGGCTTGTCCGATAACCAACCGCTCAACCAAATCAAATTCCGGGTCGGTCGTAATAAGCGGGCTTGGGTCAATAATTGTGTGAGCATCGGTTTGTCTTCTTGTTTTCTGGAGCCACTCGAGTCAACTGGAATCTATTTCATCTATGCTGCTTTGTACCAGTTGGTGAAGCACTTCCCTGACACGTCGTTTGACCCCCGGCTTTCAGACGCATTTAACGCTGAAATTGTACACATGTTTGATGACTGCCGCGACTTTGTTCAAGCTCACTATTTTACCACTTCACGCGACGACACGCCTTTCTGGCTCGCCAACCGTCACGACCTCCGCCTCTCCGATGCCATCAAGGAAAAAGTGCAGCGCTACAAGGCAGGTCTCCCCCTTACGACGACCTCTTTCGACGACAGCACCTATTACGAAACCTTCGATTACGAGTTCAAGAATTTTTGGTTGAACGGTAATTACTATTGTATCTTCGCGGGACTTGGAATGTTGCCAGATCGTTCACTCCCCCTCCTTCAACACCGCCCCGAATCCATCGAGAAGGCAGAAGCAATGTTTGCTTCAATTCGCCGCGAGGCTGAACGTCTGCGGACTTCCCTGCCAACGAACTATGATTACCTCCGTTCCCTGCGTGATGGCGACGCCGGTCTTTCTCGGGGCCAACGGGGACCTAAATTGGCTGCTCAAGAATCGCTT**TAA**aagcttGGCTGTTTTGGCGG |

Red underlined ATG means ORF (open reading frame) start codon; blue underlined TAA means ORF stop codon.

**Table S5.** Codon-optimized nucleotide sequence of *rebHF* from *Lechevalieria aerocolonigenes.*

| Codon-optimized *rebHF* ORFs from *L. erocolonigenes* (1.593 kb *rebH*; 0.513 kb *rebF*) |
| --- |
| **ATG**TCTGGCAAGATCGACAAAATTTTGATTGTAGGCGGCGGAACGGCGGGCTGGATGGCTGCCTCGTATTTGGGCAAAGCCCTGCAAGGAACCGCCGACATCACGCTTTTGCAGGCACCCGACATCCCCACCCTGGGTGTTGGTGAGGCAACTATTCCTAACCTGCAAACCGCATTCTTTGATTTTTTGGGCATCCCTGAAGACGAATGGATGCGCGAATGCAACGCTTCCTATAAAGTTGCAATCAAGTTTATCAACTGGCGCACGGCGGGCGAGGGAACCTCAGAGGCTCGTGAGTTGGATGGAGGTCCGGATCATTTCTATCATTCATTTGGTCTCTTGAAATATCATGAACAAATTCCATTGTCCCACTATTGGTTCGACCGGAGCTACCGTGGAAAGACCGTCGAGCCTTTCGACTACGCATGCTACAAAGAGCCTGTAATTTTGGATGCAAATCGGTCACCTCGGCGCCTTGATGGCAGCAAGGTCACTAACTATGCTTGGCACTTTGATGCGCACTTGGTGGCCGACTTCCTGCGTCGTTTCGCAACCGAAAAACTGGGTGTGCGTCATGTAGAAGATCGTGTAGAGCATGTCCAGCGCGACGCTAATGGCAACATCGAGAGCGTTCGGACCGCTACTGGACGCGTGTTCGACGCCGATTTGTTCGTTGATTGCTCGGGATTCCGCGGACTGCTTATCAACAAAGCGATGGAAGAACCTTTCTTGGATATGTCAGATCATCTTCTCAATGACTCCGCCGTTGCTACCCAAGTTCCGCACGATGACGACGCGAATGGTGTCGAACCCTTCACGTCCGCCATCGCTATGAAGTCCGGATGGACTTGGAAAATCCCTATGTTGGGCCGCTTTGGTACTGGTTATGTGTACTCTAGCCGGTTCGCGACCGAGGATGAGGCTGTTCGTGAGTTCTGCGAGATGTGGCATCTCGACCCTGAGACGCAGCCACTGAACCGCATCCGTTTTCGCGTAGGACGTAACCGTCGCGCATGGGTTGGTAATTGCGTCTCGATTGGTACGTCATCCTGTTTTGTTGAACCACTGGAGTCAACCGGTATCTACTTTGTATACGCTGCCCTTTACCAGCTCGTAAAACACTTTCCGGATAAATCTCTGAATCCAGTTCTTACGGCTCGCTTTAACCGGGAAATCGAGACGATGTTCGATGACACCCGTGATTTTATCCAGGCCCACTTTTATTTCAGCCCCCGCACTGATACGCCTTTTTGGCGCGCGAACAAAGAACTTCGGTTGGCAGATGGAATGCAAGAGAAAATTGACATGTATCGTGCGGGAATGGCTATCAATGCACCTGCGTCAGACGATGCACAACTCTACTACGGTAATTTTGAAGAGGAATTTCGGAATTTTTGGAACAATTCTAACTATTACTGCGTCCTCGCGGGTCTCGGTCTTGTACCTGATGCGCCGTCACCTCGTCTTGCTCACATGCCACAAGCCACGGAGTCGGTAGATGAAGTGTTTGGTGCTGTAAAGGATCGGCAGCGGAACCTCTTGGAGACGCTGCCATCCCTGCATGAGTTCTTGCGCCAACAGCACGGACGT**TAA**TACGAAAGGAGACAATTG**ATG**ACTATTGAGTTTGACCGCCCCGGAGCGCATGTAACTGCTGCCGACCATCGCGCATTGATGTCACTCTTCCCGACGGGCGTGGCAGTTATCACGGCTATCGATGAAGCGGGCACTCCTCACGGTATGACTTGCACTAGCCTGACGTCGGTTACTTTGGACCCGCCAACCCTCCTGGTATGTCTGAATCGCGCCTCTGGTACGCTGCACGCAGTACGTGGAGGACGCTTCGGCGTAAACCTGTTGCACGCTCGCGGACGGCGTGCGGCAGAAGTCTTTTCAACCGCAGTACAGGACCGCTTCGGTGAAGTCCGGTGGGAGCACTCTGATGTAACCGGTATGCCCTGGTTGGCTGAGGACGCTCACGCATTTGCAGGTTGCGTAGTCCGGAAGTCCACTGTAGTGGGTGACCATGAGATCGTATTGGGCGAGGTCCACGAAGTTGTACGTGAACACGATTTGCCCTTGCTTTACGGTATGCGTGAATTTGCCGTGTGGACGCCGGAAGGC**TAA** |

Red underlined ATG means ORF start codon; blue underlined TAA means ORF stop codon.

**Table S6.** Codon-optimized nucleotide sequence of *prnA* from *Serratia plymuthica*

| Codon-optimized *prnA* ORF from *S. plymuthica* (1.611 kb) |
| --- |
| GAAGTCCAGGAGGAgaattc**ATG**TCCAAGCCAATTAAGAACATTGTTATTGTCGGCGGCGGCACGGCCGGCTGGATGTCTGCAAGCTACCTTGTTCGAGCCCTTCAGCAGCAGGCCAACATTACTCTTATTGAAAGCGCCGCCATTCCCCGAATTGGCGTCGGCGAGGCCACCATTCCATCACTTCAGAAGGTCTTCTTCGACTTCCTTGGAATACCAGAGCAGGAGTGGATGCCCCAGGTCAACGGCGCCTTCAAGGCAGGCATTAAGTTCGTCAACTGGCGGAAGAGCCCCGACCATAGCCGCAACGACTACTTCTACCACCTTTTCGGCAACGTCCCATCCTGCGACGGCGTCCCACTGACCCACTACTGGCTTCGCAAGCGCGAACAGGGCTTCCAGCAGAGCATGGCGTACGCGTGCTACCCACAGCCCGGAGCCCTTGACGGCAACCTGGCGCCCTGCCTGCATGACGGCACCCGTCAGATGTCTCATGCCTGGCATTTCGACGCCCATCTGGTCGCAGACTTCCTTCAGCGCTGGGCAGTTGAACGCGGGGTCAACCGCGTCGTTGACGAGGTTGTTGAGGTTAACCTGAACGACCATGGCTTCATTTCTTCCCTGCTTACCAAGGAGGGCCGAAAGCTGGAGGCCGACCTGTTCATTGACTGCTCTGGCATGCGAGGACTTCTTATTAACCAGGCACTGAAGGAGCCCTTCATTGACATGTCTGACTACCTGCTGTGCGACTCCGCGGTTGCATCCGCAGTACCCAACGACGACGCCCAAGTCGGAGTTGAACCCTACACCTCTGCCATTGCAATGAACAGCGGATGGACCTGGAAGATTCCAATGCTGGGCCGATTCGGCTCCGGCTACGTTTTCAGCTCCAAGTTCACCTCTCGCGACCAGGCAACCACCGACTTCCTTAACCTTTGGGGCCTTAGCGACAACCAGCCACTTAACCAGATTAAGTTCCGAGTTGGACGCAACAAGCGAGCCTGGGTTAACAACTGCGTTTCTATTGGACTGAGCAGCTGCTTCCTTGAGCCCCTGGAAAGCACTGGAATTTACTTCATTTACGCCGCCCTTTACCAACTTGTCAAGCATTTCCCCGACACCAGCTTCGACCCACGACTTGCAGACGCCTTCAACGCAGAGATTACCTACATGTTCGACGACTGCCGGGACTTCGTTCAGGCCCATTACTTCACCAGCAGCCGCGAAGACACTCCATTCTGGCTTGCCAACCGACATGACCTGCGACTTAGCGACTCTATTAAGGAGAAGGTTGAGCGCTACAAGGCGGGACTGCCACTGACGACCACTAGCTTCGACGACTCTACTTACTACGAGACCTTCGACTTCGAgTTCAAGAACTTCTGGCTTAACGGAAACTACTACTGCATTTTCGCAGGCCTTGGCATGCTGCCCGACCGAAGCCTGCCACTTCTTCAACTGCGGCCAGAGAGCATTGAAAAGGCAGAGGCCATGTTCGCACGCATTCAGCGTGAGGCAGAGCGTCTGCGAGCCTCCCTGCCAACTAACTACGACTACCTGCGAAGCCTGCGCGGAGGCGACGCCGGACTGTTCCGGCCCGGACCAACTCCCGCCAGCCCAGAGTCCCTG**TGA**aagcttAGAGTAGGGAACTG |

Red underlined ATG means ORF start codon; blue underlined TGA means ORF stop codon.

**Table S7.** Codon-optimized nucleotide sequence of *prnA* from *Serratia grimesii*.

| Codon-optimized *prnA* ORF from *S. grimesii* (1.611 kb) |
| --- |
| GAAGTCCAGGAGGAgaattc**ATG**TCCAAGCCAATTAAGAACATTGTTATTGTCGGCGGCGGCACGGCCGGCTGGATGTCTGCAAGCTACCTTGTTCGAGCCCTTCAACAGCAGGCCAACATTACTCTTATTGAAAGCGCCACTATTCCCCGAATTGGAGTCGGAGAGGCCACCATTCCATCACTTCAGAAGGTCTTCTTCGACTACCTTGGGATACCAGAACAGGAGTGGATGCCCCAGGTCAACGGCGCCTTCAAGGCAGGCATTAAGTTCGTCAACTGGCGGACTAGCCCCGACCATAGCTCCAACGACTACTTCTACCACCTTTTCGGCAACGTCCCATCCTGCGACGGCGTCCCACTTACCCATTACTGGCTGCGCAAGCGCGAACAGGGATTCCAGCAGAGCATGGTTTACGCATGCTACCCACAGCCCGGAGCCCTTGACGGCAACCTGGCACCATGCCTGCACGACGGCACCCGTCAGATGTCTCATGCCTGGCATTTCGACGCCCATCTTGTCGCAGACTTCCTTAAGCGCTGGGCAGTTGACCGCGGAGTCAACCGGGTCGTTGACGAGGTTGTCGAGGTTAACCTGAACGAACATGGATTCATTTCTTCCCTGCTTACCAAGGAGGGCCGAAAGCTGGAGGCCGACCTGTTCATTGACTGCAGCGGCATGCGAGGACTTCTTATTAACCAGGCGCTGAAGGAGCCCTTCATTGACATGTCTGACTACTTACTTTGCGACTCCGCCGTTGCCACGGCAGTACCCAACGACGACGCCCAAGCCGGAGTTGAACCCTACACCTCTAGCATTGCAATGAACAGCGGATGGACCTGGAAGATTCCAATGCTGGGACGATTCGGCTCCGGCTACGTTTTCAGCTCCAAGTTCACCTCTCGCGACCAGGCGACCACCGACTTCCTTAACCTTTGGGGCCTTAGCGACAACCAGCCACTTAACCAGATTAAGTTCCGAGTTGGACGCAACAAGCGGAGCTGGGTTAACAACTGCGTTAGCATTGGACTGGCCAGCTGCTTCCTTGAGCCCCTGGAAAGCACTGGAATTTACTTCATTTACGCCGCCCTTTACCAACTTGTCAAGCATTTCCCCGACACCAGCTTCGACCCACGACTTGCAGACGCCTTCAACGCAGAAATTACCTACATGTTCGACGACTGCCGGGACTTCGTTCAAGCCCATTACTTCACGAGCAGCCGCGAAGACACTCCATTCTGGCTTGCCAACCAGCATGACCTGCGACTTTCTGACTCTATTAAGGAGAAGGTTGAGCGCTACAAGGCCGGACTGCCACTTACCACCACTAGCTTCGACGACTCTACTTACTACGAGACCTTCGACTTCGAgTTCAAGAACTTCTGGCTTAACGGGAACTACTACTGCATTTTCGCAGGCCTTGGCATGCTGCCCGACCGAAGCCTGCCACTTCTTCAACTTCGGCCAGAGAGCATTGAAAAGGCGGAGGTCATGTTCGCACGCATTCAGCGTGAGGCAGAGCGTCTGCGAGCCTCCCTGCCAACTAACTACGACTACCTGCGAAGCCTGCGCGACGGCGAAGTCGGACTGAGCCGGCCCGGACCAACTCCCGCCAGCCCAGAGTCCCGA**TGA**aagcttAGAGTAGGGAACTG |

Red underlined ATG means ORF start codon; blue underlined TGA means ORF stop codon.

**Table S8.** Codon-optimized nucleotide sequence of *prnF* from *P. fluorescens.*

| Codon-optimized *prnF* ORF from *P. fluorescens* (0.561 kb) |
| --- |
| GAAGTCCAGGAGGAgaattc**ATG**AATGCGGCAACCGAGACCAAAGTACACGACCTTCTTGACGCAGAGGGCCGCGACGTTCGCGACGCACGAGAGCTGCGCAACGTGCTGGGACAGTTTGCGACCGGAGTGACCGTAATCACCACCCGCACCGCGGATGGCCGCAACGTTGGAGTGACAGCAAACTCTTTCTCTAGTCTGTCTCTGAGTCCAGCCCTGGTGCTTTGGTCCCTGGCGCGCACAGCGCCATCCCTGAAGGTTTTTTGCTCTGCCTCCCACTTCGCAATCAACGTACTGGGCGCCCACCAGCTTCACCTGTCTGAGCAGTTCGCGCGTGCGGCGGCAGATAAGTTCGCAGGAGTAGCGCATTCTTATGGCAAGGCCGGGGCACCAGTGCTGGATGACGTGGTGGCGGTGCTGGTGTGCCGCAACGTTACCCAGTACGAGGGCGGAGATCACCTGATCTTCATAGGCGAGATCGAGCAATACCGCTACTCCGGAGCGGAGCCACTGGTTTTCCATGCGGGCCAGTACCGTGGATTAGGATCCAATAGAGCGGAGTCCGTTCTTAAGCACGAG**TGA**aagcttAGAGTAGGGAACTG |

Red underlined ATG means ORF start codon; blue underlined TGA means ORF stop codon.

**Table S9.** Codon-optimized nucleotide sequence of a putative *prnF* from *S. plymuthica*.

| Codon-optimized a putative *prnF* ORF from *S. plymuthica* (0.567 kb) |
| --- |
| GAAGTCCAGGAGGAgaattc**ATG**AGCGACCAAGCACTAAAGATAGTTACCCTGCTTGGATCCCTGCGTAAGGGAAGCTACAACGCAATGGTCGCACGGGCCCTGCCCGGCCTTGCACCCCAGGGAGTCATTATTGAAGCCCTTCCCAGCATTCGTGACATTCCACTGTACGACGCAGACGTCCAACAAGACGAAGGATTCCCCGCAAGCGTTGAGGCGATTGCAGCCCAAATTCGTCAGGCGGACGGCGTCATTATTGTCACCCCAGAGTACAACTACAGCGTCCCAGGCGGGCTTAAGAACGCAATTGACTGGCTTTCTCGCCTACCAAACCAGCCACTGGCAGGAAAGCCAGTAGCCATTCAGACCTCCAGCATGGGACCCATTGGCGGCGCCCGTTGCCAATACCACCTGCGCCAGATTCTGGTCTTCCTTGACGCAATGGTCATGAACAAGCCAGAGTTCATGGGCGGAGTTATTCAGAACAAGGTCGACGCCCAGGCAGGACAACTTGTTGAACAAGGCACTCTGGACTTCCTGACCGGCCAGCTTTCTGCGTTCTCCGACTACATTCGCCGCCTGAAG**TAA**aagcttAGAGTAGGGAACTG |

Red underlined ATG means ORF start codon; blue underlined TAA means ORF stop codon.

**Table S10.** Codon-optimized nucleotide sequence of a putative *prnF* from *S. grimesii*.

| Codon-optimized a putative *prnF* ORF from *S. grimesii* (0.567 kb) |
| --- |
| GAAGTCCAGGAGGAgaattc**ATG**AGCGACCAAGCGCTAAAGATTGTTACGCTGCTTGGATCCCTGCGCAAGGGCAGCTACAACGCAATGGTTGCCCACGCACTGCCCGGCCTTGCCCCACAAGGCGTTACCATTGAAGCCCTGCCCAGCATTCGTGACATTCCACTATACGACGCAGACATGCAACAGGAAGAGGGATTCCCCGCGACTGTCGAAGCAATTGCAGAGCAAATTCGCCAAGCGGACGGAGTCATTATTGTAACCCCAGAATACAACTACAGCGTACCAGGCGGGCTAAAGAACGCGATTGACTGGCTTAGCCGCCTGCCCAACCAACCCCTAGCAGGAAAGCCCGTCGCAATTCAGACCTCATCTATGGGGCCAATTGGCGGGGCGCGCTGCCAGTACCATCTGCGCCAAATTCTGGTCTTCCTTGACGCGATGGTCATGAACAAGCCAGAgTTCATGGGAGGCGTCATTCAAAACAAGGTCGACGCCCAGACTGGAGAACTTATTGACCAAGGCACTCTGGACTTCCTGACCGGCCAACTGAGCGCCTTCTCCGACTACATTCGCCGCGTCAAG**TAA**aagcttAGAGTAGGGAACTG |

Red underlined ATG means ORF start codon; blue underlined TAA means ORF stop codon.

**Table S11.** Codon-optimized nucleotide sequence of *prnB* from *P. fluorescens* BL915

| Codon-optimized *prnB* ORF from *P. fluorescens* (1.086 kb) |
| --- |
| GAAGTCCAGGAGGAgaattc**ATG**GAACGGACGTTGGACCGTGTAGGAGTGTTTGCAGCGACTCATGCAGCGGTAGCTGCGTGTGACCCTCTCCAGGCTCGCGCTCTTGTCCTCCAACTGCCTGGTCTTAACCGCAATAAAGACGTACCCGGTATCGTAGGTCTTCTGCGGGAGTTCCTCCCTGTACGGGGCCTTCCGTGTGGTTGGGGCTTCGTAGAAGCGGCTGCAGCTATGCGGGATATTGGCTTTTTTTTGGGCTCCCTTAAACGCCATGGACACGAACCCGCCGAGGTCGTACCTGGCCTTGAGCCGGTCCTTCTGGACCTGGCCCGGGCAACGAATTTGCCACCACGGGAAACTCTTTTGCATGTAACTGTTTGGAATCCAACGGCTGCGGACGCCCAGCGCAGCTACACTGGCCTGCCTGACGAGGCTCATCTGCTCGAATCAGTTCGGATCTCTATGGCAGCCTTGGAAGCGGCGATCGCGCTCACTGTAGAACTGTTTGACGTATCCCTGCGCAGCCCTGAATTTGCCCAACGCTGCGACGAGCTGGAAGCGTATCTTCAAAAGATGGTAGAGTCTATTGTTTACGCCTACCGTTTCATCAGCCCCCAAGTATTTTATGACGAGTTGCGCCCTTTTTATGAGCCAATCCGCGTAGGTGGTCAAAGCTACCTCGGTCCCGGTGCCGTGGAAATGCCTCTGTTCGTGCTTGAGCATGTGCTTTGGGGCTCACAGAGCGATGACCAGACCTATCGTGAATTTAAAGAAACGTATCTCCCTTACGTCTTGCCTGCTTACCGGGCTGTTTACGCCCGTTTCTCGGGAGAACCGGCGTTGATTGATCGCGCTCTCGATGAGGCCCGTGCCGTTGGCACGCGTGATGAGCACGTGCGGGCTGGCCTCACGGCTTTGGAGCGTGTATTTAAAGTGCTTCTGCGGTTCCGGGCACCACACCTGAAGCTCGCTGAGCGGGCTTATGAAGTGGGACAATCCGGACCGGAGATCGGTTCAGGTGGATATGCGCCATCAATGCTGGGCGAATTGCTCACGCTGACGTATGCGGCCCGCTCTCGTGTTCGCGCAGCGCTTGATGAATCA**TAA**aagcttAGAGTAGGGAACTG |

Red underlined ATG means ORF start codon; blue underlined TAA means ORF stop codon.

**Table S12.** Codon-optimized nucleotide sequence of *prnB* from *Burkholderia contamins.*

| Codon-optimized *prnB* ORF from *B. contamins* (1.086 kb) |
| --- |
| GAAGTCCAGGAGGAgaattc**ATG**GAGCGTGCGCTTGGACGCGCTCGGGCTTTCGCCGCAACTCATGCCGCAGTAGCTGCTTGTGACCCTCTGCGGGCACGTGCCCTTGTATTGCAGCTCCCTGCATTGAACCGTAAGGATGACGTGCCGGGAATTGTGGGATTGTTGCGCGAGTTTCTTCCCACGCGTGGCGTTCCATCTGGCTGGGGATTCGTTGAGGCAGCGGCTGCCATGCGTGATATTGGATTCTTCCTTGGTAGCCTCAAACGGCACGGCCACGAGCCGGTTGACGCGGTTCCTGGCCTGGAACCTGTTCTCCTTGATTTGGCCCGCGTAACCGATCTTCCACCTCGTGAGACGCTTTTGCATGTTACTGTGTGGAACCCGGCAACGGCGGACGCACAGCGGAGCTATACTGGCCTGGGTGACGAGGCCCATCTTTTGGAGAGCGTGCGCATCTCAATGGCATCTTTGGAAGCCGCGATCGCCTTGACGGTCGAGCTCTACGACGTTCCTTTGCGTTCGCCAGCTTTTGAGGAGGGCTGTGTCGAATTGGCCGCCCACCTGCAGAAAATGGTAGAGTCTATTGTATATGCGTACCGGTTCATTTCTCCGCAAGTGTTTTATGACGAGCTCCGGCCATTCTACGAGCCTATTCGTGTTGGTGGCCGGTCCTACCTTGGCCCAGGCGCAGTTGAGATGCCATTGTTTGTGCTCGAACACGTTCTTTGGGGCTCTCAATCAGACCATCCAGCCTATCTTGAGTTCAAGGAGACCTATCTGCCATATGTTCTTCCTGCTTTCCGCGCCATTTATGCGCGGTTCGCAGGACGTCAAGCGCTCGTAGATCGTGTGCTTGGAGAAGCACAAGCTGCGCGGGAGCGCGGCGAGCCAGTAGGAGCGGGTCTTGCAGCACTCGAGCGCATTTTCGAGATTCTGCTCCACTTCCGGGCTCCGCATCTTAAGCTGGCCGAACGGACGTACGCCGCGGGCCAAACTGGACCAACCATCGGTTCTGGAGGATACGCGCCGTCGATGCTTGGTGACCTTCTCACTCTTACCCGCGACGCTCGGTCCCGTCTTCATGCCGTACTCGCGGAAACT**TAA**aagcttAGAGTAGGGAACTG |

Red underlined ATG means ORF start codon; blue underlined TAA means ORF stop codon.

**Table S13.** Codon-optimized nucleotide sequence of *prnB* from *S. plymuthica* PR1-2C.

| Codon-optimized *prnB* ORF *S. plymuthica* (1.086 kb) |
| --- |
| GAAGTCCAGGAGGAgaattc**ATG**GAGCGCGCACATGACCGAGCGTGCGTATTCGCCGCAACTCATGCGGCAGTCGCCGCATGCGACCCACTGCAGGCCCGCAGCCTTGTTCTGCAACTGCCAGACCTGAACCGTAACAAGGACGTCCCCGGCATTGTCGGCCTGCTGCGCGAGTTCCTTCCAGTCCGCGGCGTCCCCTCTGGGTGGGGCTTCGTTGAAGCAGCAGCAGCCATGCGAGACATTGGATTCTTCCTGGGAAGCCTTAAGCGACACGGGCATGAGCCCGTCGACGTCGTCCCCGGACTTGAGCCAGTCCTGCTTGACCTGGCCCGCATGACCGACCTGCCACCACGCGAGACTCTTCTGCACGTCACTGTTTGGAACCCCGCCGCCGCAGACGCCCAACGATCCTACACCGGACTTTCCGACGAAGCCCATCTGCTTGAGTCCGTCCGCATTAGCATGGCCGCACTTGAGGCCGCAATTGCCGTCACCGTTGAGCTGCATGACGTCCCCCTGAGGAGCCCCGCCTTCGCCCAAGGATGCGACGAGCTGGCCGCATACCTGCAAAAGATGGTTGAAAGCATTGTTTACGCCTACCGCTTCATTAGCCCACAGGTTTTCTACGACGAGCTTCGCCCCTTCTACGAACCCATTCGCGTTGGAGGCCAGAACTACCTTGGCCCCGGAGCAGTAGAAATGCCCCTTTTCGTCCTGGACCATGTTCTGTGGGGCAGCCAAAGCGACCATCAGGCGTACCGGGAgTTCAAGGAGACTTACCTGCCCTACGTCCTTCCCGCCTACCGAGCCGTTTACGCGCGATTCGCAGGAAGGCCAAGCCTTGTTGACCGTGCCATTGGCGAGGCCCGGGCCGCAGGAGCACAGGGCGAATCTGTTCGAGCAGGACTGGCCGCACTTAACCGAGTTTTCGTCGTTCTGCTGCGCTTCCGAGCGCCCCACGTTCAACTTGCCGAGCGAGTCTACGAAGTTGGACGGTCCGGCCCAGCGATTGGCTCAGGAGGATACGCCCCCTCCATGCTTGGCGACCTGGCAACTCTTACTCTTGCAGCCCGCTCTCGCATTCGCACCGCGCTTAACGAGTCT**TAG**aagcttAGAGTAGGGAACTG |

Red underlined ATG means ORF start codon; blue underlined TAG means ORF stop codon.

**Table S14.** Codon-optimized nucleotide sequence of *prnB* from *S. grimesii.*

| Codon-optimized *prnB* ORF from *S. grimesii* (1.083 kb) |
| --- |
| GAAGTCCAGGAGGAgaattc**ATG**GAGCGCGCACTGGACCGAGCGTGCGTATTCGCAGCAACTCATGCGGCAGTCGCGGCATGCGACCCACTGCAGGCGCGCAGCCTTGTTCTTCAACTGCCAGGCCTGAACCGTAACAAGGACGTCCCCGGAATTGTTGGCCTGCTGTGCGAGTTCCTTCCAGTCCGCGGCGTCCCCTCTGGATGGGGATTCGTTGAAGCAGCAGCAGCCATGCGAGACATAGGATTCTTCCTGGGAAGCCTTAAGCGACATGGGCATGAACCCGTCGACGTCGTCCCCGGACTTGAGCCAGTCCTGCTTGACCTGGCCCGCATGACCGACCTGCCACCACGTGAGACTCTTCTGCACGTCACTGTTTGGAACCCCGCGGCCGCAGACGCCCAACGATCATACACCGGACTTTCCGACGAAGCGCATCTGCTTGAGTCCGTCCGCATTAGCATGGCCGCACTTGAAGCCGCAATTACTGTCACCGTTGAGCTGCATGACGTACCCCTTAGGAGCCCCGCCTTCGCCCAAGGATGCGACGACCTGGCGGCATACCTGCAAAAGATGGTTGAAAGCATTGTTTACGCGTACCGCTTCATTAGCCCACAGGTTTTCTACGACGAGCTTCGCCCCTTCTACGAACCAATTCGCGTTGGAGGACAAAACTACCTTGGCCCCGGCGCGGTAGAAATGCCCCTTTTCGTCCTGGACCATGTTCTGTGGGGCAGCCAAAGCGACCATCCAGCGTACCAAGAgTTCAAGGAGACTTACCTGCCCTACGTACTTCCCGCCTACAGGGCCGTTTACGCACGATTCGCGGGAAAGCCAAGCCTTGTTGACCGCGTCATTGGCGAGGCCCGGGTAGGAGCCCAAGGCGAACCCGTTCGAGCAGGACTGGCCGCCCTTAACCGAATTTTCGTCATTCTGCTGCGCTTCCGAGCGCCCCACGTTCAACTTGCCGAGCGAGTCTACGAAGCAGGACGGTCCGGCCCAGCGATTGGCTCAGGGGGATACGCCCCCTCCATGCTTGGCGACCTGGTTACTCTTACTCTTGCAGCCCGCTCTCGCATTCGCACCGCGCTTAACGAGTTC**TAG**aagcttAGAGTAGGGAACTG |

Red underlined ATG means ORF start codon; blue underlined TAG means ORF stop codon.

**Table S15.** Codon-optimized nucleotide sequence of *prnC* from *P. fluorescens* BL915

| Codon-optimized *prnC* gene ORF from *P. fluorescens* (1.704 kb) |
| --- |
| GAAGTCCAGGAGGAgaattc**ATG**ACTCAAAAGTCACCTGCGAATGAACACGATTCCAATCACTTTGATGTTATCATCCTGGGTTCGGGTATGTCGGGCACTCAGATGGGAGCTATCCTCGCCAAACAACAGTTTCGCGTCCTTATTATCGAGGAATCATCACACCCGCGTTTTACGATCGGCGAAAGCTCGATTCCTGAGACGAGCCTGATGAATCGCATTATCGCTGATCGCTATGGCATTCCTGAGTTGGACCATATCACTTCGTTTTACAGCACGCAGCGTTACGTGGCGAGCAGCACTGGTATCAAACGTAATTTCGGATTCGTGTTTCACAAACCGGGCCAAGAGCATGATCCAAAGGAATTTACTCAATGTGTGATCCCTGAGCTGCCATGGGGCCCGGAATCCCATTACTATCGCCAGGACGTAGATGCATATCTGCTTCAAGCCGCCATTAAATACGGCTGCAAAGTGCATCAAAAGACGACTGTAACTGAGTACCATGCGGATAAAGATGGCGTAGCGGTCACCACCGCCCAGGGCGAACGTTTCACTGGTCGCTATATGATTGATTGTGGAGGTCCCCGTGCTCCATTGGCCACTAAGTTTAAGTTGCGTGAAGAGCCGTGTCGTTTTAAGACTCATTCGCGCTCTCTGTACACTCACATGTTGGGAGTGAAACCCTTCGATGATATCTTCAAGGTAAAAGGTCAGCGTTGGCGGTGGCACGAAGGTACGCTCCACCATATGTTCGAGGGAGGATGGTTGTGGGTAATCCCGTTTAATAATCACCCCCGTAGCACCAATAATCTCGTGTCGGTGGGCTTGCAGCTGGATCCACGGGTCTACCCTAAGACCGATATCTCCGCGCAGCAAGAGTTTGATGAATTTCTGGCTCGCTTCCCATCAATTGGTGCTCAATTTCGGGATGCGGTCCCTGTGCGGGACTGGGTGAAGACGGATCGCCTGCAATTTTCATCTAACGCGTGTGTAGGCGACCGCTATTGTCTTATGTTGCATGCAAACGGTTTTATCGACCCTCTTTTCAGCCGGGGACTGGAGAACACCGCGGTAACCATTCATGCGTTGGCGGCTCGGCTTATCAAGGCGCTGCGCGATGATGACTTTAGCCCTGAGCGCTTCGAGTATATCGAGCGCTTGCAGCAGAAATTGTTGGATCACAATGATGATTTTGTCTCTTGTTGCTACACCGCCTTTTCAGACTTTCGCTTGTGGGATGCATTCCACCGGTTGTGGGCTGTAGGTACGATTCTGGGCCAGTTTCGCCTTGTCCAAGCACACGCACGCTTTCGGGCCTCCCGTAACGAAGGCGACCTGGACCACTTGGACAACGACCCGCCCTATCTCGGTTACCTGTGCGCCGACATGGAAGAGTATTATCAATTGTTCAACGATGCAAAAGCGGAGGTGGAGGCGGTAAGCGCGGGCCGTAAGCCCGCAGATGAGGCCGCCGCTCGGATCCACGCCCTTATCGATGAGCGGGACTTCGCTAAGCCAATGTTTGGCTTTGGCTACTGCATCACGGGAGACAAGCCACAGTTGAACAACTCCAAATACAGCCTTCTCCCTGCAATGCGGCTGATGTATTGGACGCAAACCCGCGCACCAGCGGAGGTGAAAAAATATTTTGATTACAACCCAATGTTCGCCTTGCTTAAGGCGTATATTACGACTCGCATTGGCTTGGCGTTGAAAAAG**TAA**aagcttAGAGTAGGGAACTG |

Red underlined ATG means ORF start codon; blue underlined TAA means ORF stop codon.

**Table S16.** Codon-optimized nucleotide sequence of *prnC* from *B. contamins.*

| Codon-optimized *prnC* ORF from *B. contamins* (1.701 kb) |
| --- |
| GAAGTCCAGGAGGAgaattc**ATG**ACGCAGAAGAGCATCGCTAATGAACGGGATAATCACCACTTTGACGTCATTATCCTTGGATCTGGTATGTCAGGCACTCAAATGGGCGCTATCCTGGCGAAGCAAAAGTTCCGTGTACTGATCATCGAAGAATCGTCACACCCGCGTTTCACTATTGGCGAGTCGTCTATCCCGGAAACTAGCCTTATGAACCGGATCATTGCAGATCGTTACGGCATCCCTGAGTTGGATCATATTACTTCTTTCTATGCAACGCAGCGCTACGTCGCTTCGTCAACCGGTATTAAGCGCAATTTCGGTTTCGTGTTCCATAAGCCTGGAGAGGAGCACGACCCTAAAGAGTTCACCCAATGCGTTATCCCTGAATTGCCCTGGGGCCCCGAGTCACACTATTATCGTCAGGATGTGGACGCGTACCTTCTTCAGGCTGCTATTAAGTATGGCTGCACTGTCCGGCAGCGGACCTCCGTGACTGATTATCACGCGGATAAAGATGGCGTAGCCGTTGCGACCGCACAAGGAGAGCGCTTTACTGGTCGCTACATGATCGATTGTGGAGGTCCGCGCGCACCACTTGCGACTAAGTTTAATCTTCGCGAAGAGCCATGTCGTTTCAAGACCCACTCCCGGTCTCTTTATACGCACATGCTCGGAGTGAAGCCTTTCGATGATATTTTTAAGGTTAAAGGACAGCGCTGGCGGTGGCACGAGGGAACCCTCCATCACATGTTCGAGGGTGGATGGCTGTGGGTCATTCCTTTCAACAACCATGCTCGGTCTACCAATAATCTGGTCTCTGTGGGACTGCAACTTGACCCGCGGGTCTACCCCAAGACTGATATTCCTGCTCAGCAAGAATTTGATGAGTTCCTTGCCCGCTTTCCTTCTATTGGCGCGCAATTCCGGGACGCAGTACCCGTGCGTGACTGGGTTAAGACGGATCGTCTTCAGTTTTCATCCCGTGCGTGTGTTGGTGATCGTTATTGTTTGATGCTTCACGCGAACGGCTTCATCGATCCCCTCTTCTCACGCGGACTGGAGAATACTGCCGTCACCATTCATGCACTCGCGGCTCGGCTTATCAAGGCCCTTCGTGACGATGACTTCTCTCCTGAGCGTTTTGAGTATATCGAGCGGCTGCAGCAAAAGCTGCTTGATCATAATGACGACTTCGTGTCATGTTGTTATACGGCATTTTCGGACTTTCGCCTGTGGGACGCCTTTCACCGCCTCTGGGCTGTCGGCACCATTCTTGGCCAATTCCGTCTGGTACAAGCACACGCGCGCTTTCGCGCATCCCGCAACGAGCGGGATTTGGATCATCTGGATGACAACGCTCCCTACCTCGGCTTCCTGTGTGCCGACATGGAGGGTTACTACCAATTGTTTAACGACGCTAAAGCTGAAGTTGAAGCCGTATCCGCAGGACGCAAAACTGCTGGCGAGGCTGCGGCCCGCATCCACGTCTTGATTAACGAACGTGAATTTGCCAAGCCGATGTTTGGTTTTGGATATTGTATCACGGGTGCGAAGCCACAATTGAATAATTCTAAATATTCGCTCCTCCCCGCTATGAAACTCCTGCATTGGACTCAAACGTCGGCGCCCGCGGAGGTGAAGAAATACTTTGACTATAATCCGATGTTTGCCCTTTTGCGGGCGTACGTCACTACTCGCATTGGCCTGGCTCTGAAG**TAA**aagcttAGAGTAGGGAACTG |

Red underlined ATG means ORF start codon; blue underlined TAA means ORF stop codon.

**Table S17.** Codon-optimized nucleotide sequence of *prnC* from *S. plymuthica* PR1-2C.

| Codon-optimized *prnC* ORF from *S. plymuthica* (1.704 kb) |
| --- |
| GAAGTCCAGGAGGAgaattc**ATG**ACGCAGAAGTCCCCCGCCCATGGACGCGACAACAACCATTTCGACGTCATTATTCTTGGCAGCGGCATGTCTGGCACCCAGATGGGAGCGATTCTGGCAAAGCAGCAGTTCCGCGTCCTGATTATTGAGGAGAGCAGCCATCCACGATTCACTATTGGCGAAAGCAGCATTCCCGAGACTAGCCTGATGAACCGTATTATTGCAGACCGCTACGGCATTCCAGAGCTTGACCATATTACTAGCTTCTACAGCACTCAACGTTACGTTAGCAGCTCCACTGGCATTAAGCGCAACTTCGGCTTCGTCTTCCATAAGCCCGGCCAGGAGCATGACCCAAAGGAGTTCACTCAGTGCGTTATTCCCGAGCTTCCCTGGGGACCAGAGTCCCACTACTACCGACAAGACGTTGACGCATACCTGCTTCAAGCAGCAATTAAGTACGGATGCACTGTTCGCCAGAAGACGAAGGTCACCGAATACCATGCAGACAAGGACGGCGTTGCCGTCACCACCGCAGAGAACGAACGATTCACCGGCCGATACATGATTGACTGCGGGGGGCCCCGCGCCCCACTTGCCACCAAGTTCAACCTTCGCGAAGAGCCATGCCGCTTCAAGACTCATAGCCGCTCCCTTTACACTCATATGCTTGGAGTTAAGCCATTCGACGACATTTTCAAGGTTAAGGGACAGCGTTGGCGCTGGCATGAGGGAACCCTTCATCATATGTTCGAGGGCGGCTGGCTTTGGGTCATTCCATTCAACAACCATCCACGAAGCACCAACAACCTGGTCTCCGTTGGCCTGCAGCTTGACCCACGTGTTTACCCAAAGACTGACATTTCTGCCCAGCAAGAGTTCGACGAGTTCCTTGCCCGATTCCCATCCATTGGAGCCCAGTTCCGAGACGCAATTCCAGTCCGCGACTGGGTTAGGACCGACCGCCTGCAGTTCAGCAGCACCCGTTGCATTGGCGACCGCTACTGCCTGATGCTGCATGCCAACGGATTCATTGACCCACTTTTCTCTCGAGGACTTGAGAACACCGCGGTCACCATTCATGCCCTTGCCGCACGCCTTATTAAGGCCCTGCGCGACGACGACTTCTCTCCCGAGCGCTTCGAGTACATTGAGCGCCTGCAGCAAAAaCTTCTTGACCATAACGACGACTTCGTTTCCTGCTGCTACACTGCCTTCAGCGACTTCCGCCTGTGGGACGCCTTCCATAGGCTGTGGGCAGTTGGCACTATTCTTGGACAGTTCCGACTTGTCCAAGCACATGCCAGGTTCCGCGCCAGCCGCGACGAGGGCGACCTTGACCATCTTGACAACGACCCCCCATACCTTGGATACCTGTGCGCCGACATGGAGGGATACTACCAACTTTTCAACGACGCAAAGGCAGAGATTGAGGCCGTCTCCGCAGGACGCAAGCCAACCGGAGAGGCAGCCGCCCGAATcCATGCACTTATTGACGAACGGGACTTCGCAAAGCAGATGTTCGGCTTCGGATACTGCATTACCGGAGACAAGCCACAGCTTAACAACAGCAAGTACTCCCTGCTGCCAGCGATGAAGCTGATGCATTGGACGCAAACCTCCGCCCCAGCCGAGGTCAAGAAGTACTTCGACTACAACCCAATGTTCGCCCTGCTTAAGGCCTACATTACTACCCGCATTTCCCTTACGCGAAAGAAG**TGA**aagcttAGAGTAGGGAACTG |

Red underlined ATG means ORF start codon; blue underlined TGA means ORF stop codon.

**Table S18.** Codon-optimized nucleotide sequence of *prnC* from *S. grimesii.*

| Codon-optimized *prnC* ORF from *S. grimesii* (1.704 kb) |
| --- |
| GAAGTCCAGGAGGAgaattc**ATG**ACCCAAAATTCGCCCGCCAATGGCCGTGATAATAATCATTTCGACGTGATTATTCTTGGATCCGGTATGTCAGGCACCCAAATGGGAGCAATCCTGGCTAAGCAACAATTCCGCGTCCTTATCATTGAAGAATCGTCTCATCCGCGGTTTACGATTGGAGAATCGAGCATTCCCGAGACGTCGCTCATGAACCGGATTATCGCTGACCGCTACGGCATCCCAGAGCTCGATCACATTACGTCATTTTATTCGACTCAACGCTACGTCTCCTCGTCTACTGGTATTAAACGCAATTTCGGCTTTGTGTTTCATAAACCGGGCCAAGAACATGATCCGAAGGAGTTCACCCAGTGTGTAATCCCGGAGCTGCCGTGGGGACCCGAGAGCCATTACTACCGTCAAGATGTTGACGCTTACTTGCTTCAGGCAGCGATTAAGTACGGATGTACTGTCCGTCAGAAGACTAAGGTGACCGAATACCATGCTGATAAGGATGGCGTAGCTGTAACTACCGCAGAGGATGAACGTTTTACCGGACGCTATATGATCGATTGTGGAGGCCCTCGCGCGCCTCTTGCAACCAAGTTCAATCTTCGTGAGGAACCCTGTCGGTTCAAGACGCACTCGCGCTCTTTGTACACCCACATGCTGGGTGTAAAACCATTTGATGACATCTTTAAAGTTAAAGGCCAACGCTGGCGCTGGCATGAAGGCACGCTGCATCATATGTTCGAGGGTGGCTGGCTTTGGGTGATCCCGTTTAATAATCATCCACGGTCAACTAACAACCTTGTTTCCGTGGGCCTGCAACTCGACCCGCGGGTTTACCCTAAGACCGACATCTCAGCGCAGCAGGAATTTGACGAGTTCTTGGCACGTTTCCCCAGCATTGGTGCTCAATTCCGCGATGCAGTGCCAGTTCGCGATTGGGTGAAAACCGACCGTTTGCAGTTTAGCAGCACTACCTGCATTGGTGATCGTTATTGTCTGATGTTGCATGCAAATGGCTTTATTGATCCACTCTTCTCACGCGGCCTCGAAAATACGGCGGTGACCATCCACGCTCTGGCGGCTCGTTTGATTAAGGCACTGCACGATGATGACTTCTCTCCTGAGCGTTTTGAGTACATCGAACGTCTCCAGCAAAAATTGTTGGATCATAATGACGACTTCGTGTCTTGCTGCTACACCGCGTTTACGGATTTCCGCCTCTGGGACGCCTTTCACCGCCTTTGGGCCGTGGGAACCATCCTCGGCCAGTTTCGTTTGGTACAGGCACACGCACGTTTTCGGGCCTCTCGGGATGAAGGTGACCTGGATCATCTGGACAATGACCCACCTTACCTTGGCTACCTGTGTGCCGACATGGAAGGCTACTATCAACTTTTCAACGACGCGAAAGCGGAAATTGAAGCCGTGTCGGCCGGTTTGAAGCCCACCGGTGAGGCTGCTGCTCGTATCCATGCGCTCATTGATGAGCGGGACTTCGCAAAGCAGATGTTTGGATTCGGATATTGCATCACGGGCGATAAACCCCAGTTGAATAATTCCAAATATTCGTTGCTGCCCGCCATGAAGCTGATGCACTGGACTCAAACCTCGGCTCCCGCCGAAGTTAAGAAATACTTCGACTACAATCCAATGTTTGCGTTGCTGAAGGCATACATTACGACTCGCATTTCTCTTACTCGTAAAAAG**TAA**aagcttAGAGTAGGGAACTG |

Red underlined ATG means ORF start codon; blue underlined TAA means ORF stop codon.

**Fig. S1**

**Fig. S2**

**
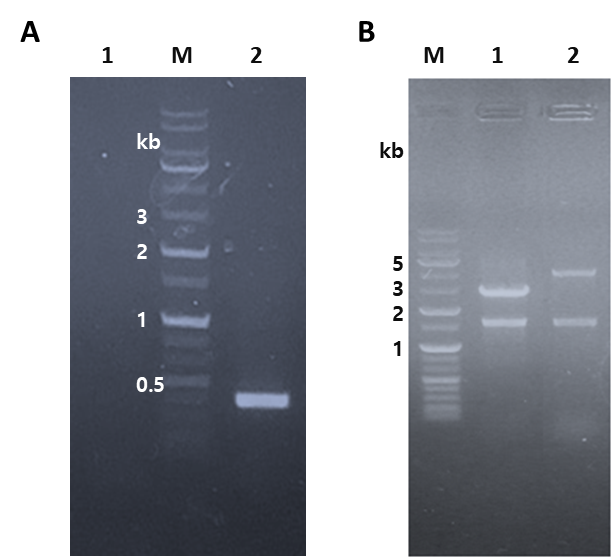
**

**Fig. S3.**


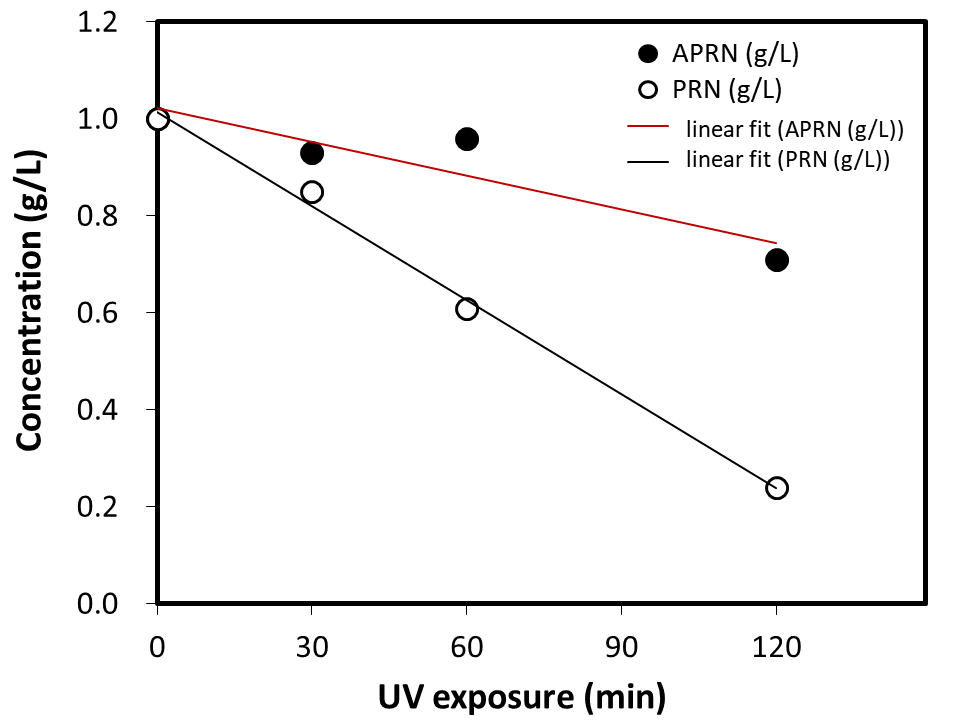


**Fig. S4**

**Fig. S5**

**Fig. S6**

**Fig. S7**

Fig. S8.


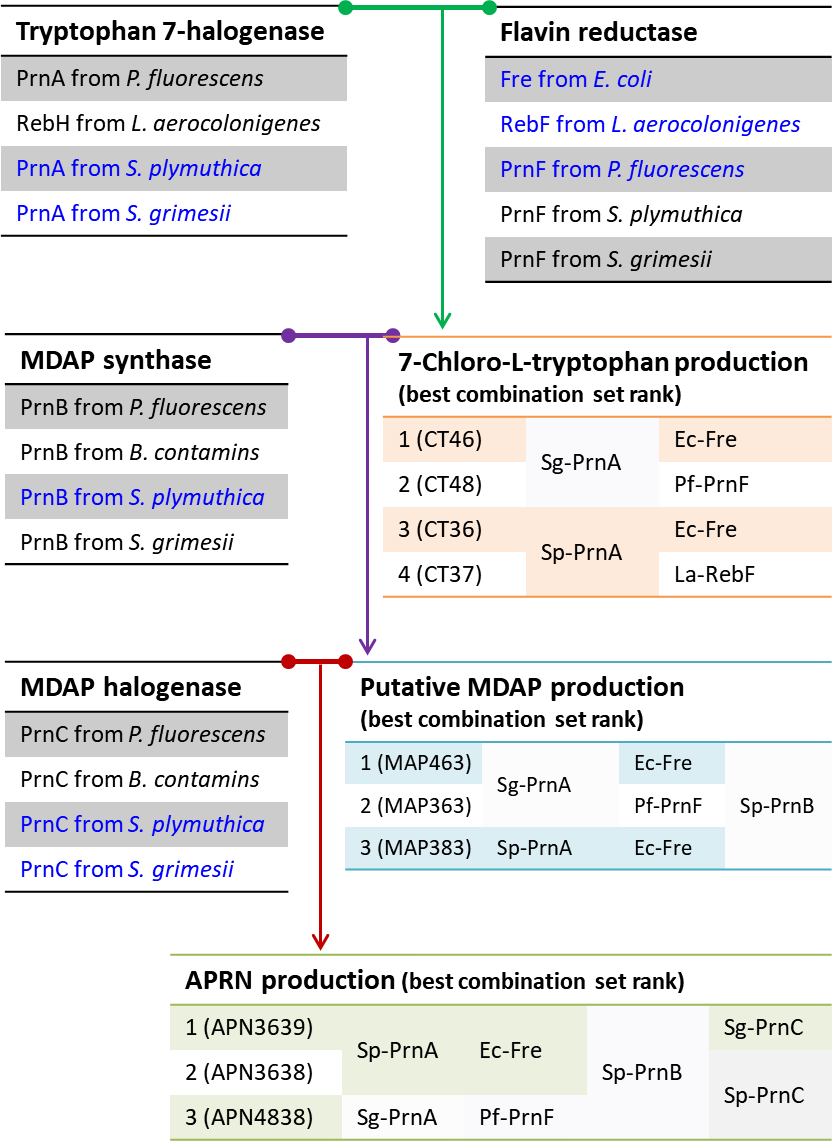

Supplement: Supplementary file 1 — Supplementary Material 1 [file 12934_2024_2424_MOESM1_ESM.docx]
